# Cardiovascular magnetic resonance physics for clinicians: part I

**DOI:** 10.1186/1532-429X-12-71

**Published:** 2010-11-30

**Authors:** John P Ridgway

**Affiliations:** 1 Department of Medical Physics and Engineering, Leeds Teaching Hospitals NHS Trust, Leeds, UK

## Abstract

There are many excellent specialised texts and articles that describe the physical principles of cardiovascular magnetic resonance (CMR) techniques. There are also many texts written with the clinician in mind that provide an understandable, more general introduction to the basic physical principles of magnetic resonance (MR) techniques and applications. There are however very few texts or articles that attempt to provide a basic MR physics introduction that is tailored for clinicians using CMR in their daily practice. This is the first of two reviews that are intended to cover the essential aspects of CMR physics in a way that is understandable and relevant to this group. It begins by explaining the basic physical principles of MR, including a description of the main components of an MR imaging system and the three types of magnetic field that they generate. The origin and method of production of the MR signal in biological systems are explained, focusing in particular on the two tissue magnetisation relaxation properties (T1 and T2) that give rise to signal differences from tissues, showing how they can be exploited to generate image contrast for tissue characterisation. The method most commonly used to localise and encode MR signal echoes to form a cross sectional image is described, introducing the concept of k-space and showing how the MR signal data stored within it relates to properties within the reconstructed image. Before describing the CMR acquisition methods in detail, the basic spin echo and gradient pulse sequences are introduced, identifying the key parameters that influence image contrast, including appearances in the presence of flowing blood, resolution and image acquisition time. The main derivatives of these two pulse sequences used for cardiac imaging are then described in more detail. Two of the key requirements for CMR are the need for data acquisition first to be to be synchronised with the subject's ECG and to be fast enough for the subject to be able to hold their breath. Methods of ECG synchronisation using both triggering and retrospective gating approaches, and accelerated data acquisition using turbo or fast spin echo and gradient echo pulse sequences are therefore outlined in some detail. It is shown how double inversion black blood preparation combined with turbo or fast spin echo pulse sequences acquisition is used to achieve high quality anatomical imaging. For functional cardiac imaging using cine gradient echo pulse sequences two derivatives of the gradient echo pulse sequence; spoiled gradient echo and balanced steady state free precession (bSSFP) are compared. In each case key relevant imaging parameters and vendor-specific terms are defined and explained.

## Background

This review is the first of two that aim to cover the basic physical principles underlying the most commonly used cardiovascular magnetic resonance (CMR) techniques. There are numerous texts and journal articles that provide excellent, in-depth explanations of MR physics and in particular CMR physics [1-5]. This review does not intend in any way to supplant these but rather to provide an overview of the key physical principles that underlie the most commonly used CMR techniques. This review begins with the basic principles of MR signal generation and image formation, outlines the principles of cardiac synchronisation and fast, breath-hold imaging. Finally, the principles behind the two most common CMR techniques; anatomical imaging using a double inversion, black-blood spin echo pulse sequence and bright blood functional cine imaging using two gradient echo-based pulse sequences are described in some detail.

## MR system components

**Figure 1:**
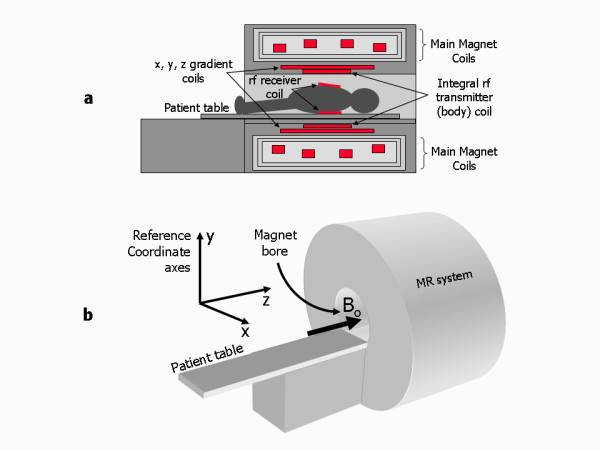
**MR system components**. a) Diagram showing the relative locations of the main magnet coils, x, y, and z gradient coils, integral rf transmitter body coil and rf receiver coils. b) Typical arrangement for a cylindrical bore MR system showing the magnet bore and the reference coordinate axes with the static B_o _field direction along the horizontal z axis.

A magnetic resonance imaging (MRI) system comprises three main electromagnetic components; a set of main magnet coils, three gradient coils and an integral radiofrequency transmitter coil (Figure 1a). These components each generate a different type of magnetic field which, when applied to a patient in combination, produce spatially encoded magnetic resonance signals that are used to form MR images. The three different types of magnetic field are defined as follows: A strong, constant magnetic field is generated by the main magnet coils. The patient is positioned for imaging within the central bore of the magnet (Figure 1b), where the strength of this field, denoted by the symbol B_o_, defines the nominal operating field strength of the particular MRI system. B_o _is measured in units of Tesla, (T) with 1 Tesla equal to approximately 20,000 times the earth's magnetic field. Nominal field strengths range from 0.2T to 3.0T for commercially produced clinical MR systems, with the most common field strength for cardiac imaging being 1.5T. A reference coordinate system of three orthogonal axes, x, y and z is used to define the magnetic field direction, with the z axis chosen to be parallel to the direction of B_o_.

A gradient magnetic field that can be rapidly switched on and off is generated by each of the three gradient coils mounted inside the main magnet (Figure 1a). Each of these gradient coils generates a magnetic field in the same direction as B_o _but with a strength that changes with position along the x, y or z directions, according to which gradient coil is used. This gradient field is superimposed onto the B_o _magnetic field so that its strength increases (or decreases) along the direction of the applied gradient field. The strength of the gradient magnetic field reflects the 'steepness' of its slope and is measured in units of millitesla per metre (mT/m).

A radiofrequency (rf) magnetic field is generated by the rf transmitter coil mounted inside the gradient coil, closest to the patient (Figure 1a). It has a much smaller amplitude than the other magnetic fields, but oscillates at a characteristic frequency in the megahertz range (hence, radiofrequency), the value of which is determined by the nominal field strength of the main magnet. The rf field is often referred to as the B_1 _field. The static magnetic field and radiofrequency field combine to generate magnetic resonance signals that are spatially localised and encoded by the gradient magnetic fields to create an MR image. For cardiac imaging, a separate rf receiver coil that is tailored to maximise signal from the heart is normally used to detect the emitted MR signals (Figure 1a).

## Generating MR signals

### Origin of the MR signal

**Figure 2:**
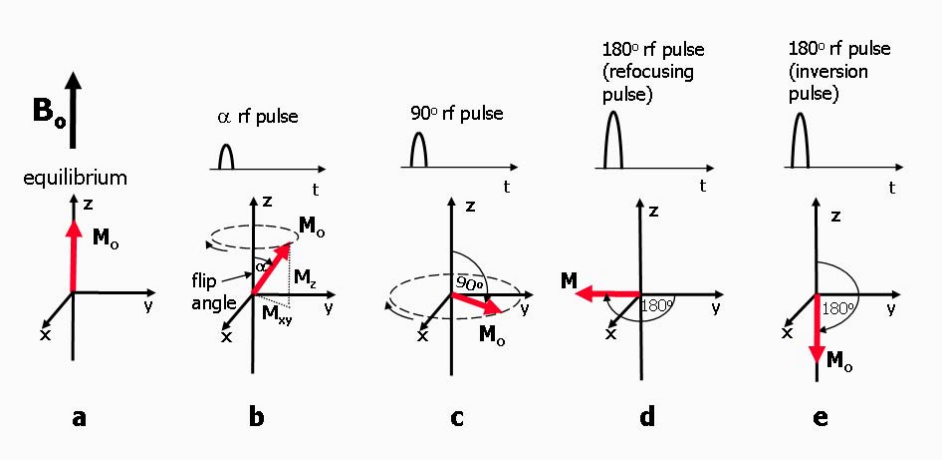
**Net Magnetisation, rf pulses and flip angle**. a) At equilibrium, the net magnetisation, M_o _is at equilibrium, aligned along the a axis. b). When an rf pulse is applied, M_o _makes an angle with the z-axis, known as the flip angle, and rotates around the axis in the direction of the curved arrow. At any instant the magnetisation can be split into two components, M_z _and M_xy_. The rotating M_xy _component generates the detectable MR signal. c) The maximum detectable signal amplitude after a single rf pulse occurs when M_o _lies entirely in the plane of the x and y axes as this gives the largest M_xy _component. This pulse has a 90° flip angle and is referred to as a 90° rf pulse or saturation pulse. d) A 180° rf refocusing pulse is usually applied while there is transverse magnetisation already rotating in the xy plane and is used to instantaneously flip the transverse component of magnetisation through 180° about an axis also rotating in the xy plane. e) A 180° inversion pulse is usually applied at equilibrium and is used to rotates the net magnetisation through 180° from the positive to the negative z axis. This is also know as a magnetisation preparation pulse and is used is the preparation scheme for black blood imaging techniques.

The primary origin of the MR signal used to generate images is either from water or fat within the patient's tissue; specifically it is from the hydrogen nuclei (consisting of a single proton) contained within free water or lipid molecules Hydrogen is one of a number of elements, including ^31^P, ^23^Na, ^13^C, whose nuclei exhibit magnetic resonance properties but the high intrinsic sensitivity and natural abundance in the form of water and lipid molecules makes it particularly favourable for imaging. Hydrogen nuclei, (single protons) possess an intrinsic property known as nuclear spin that gives rise to a small magnetic field for each proton, known as a magnetic moment. Normally the magnetic moments (spins) are randomly oriented but in the presence of the externally applied B_o _field, they tend to align either toward or against the externally applied magnetic field. An equilibrium state is quickly attained where there is a small excess of spins aligned with the field (typically just a few per million) as this is the more energetically favourable direction of alignment. The excess of proton magnetic moments combines to form a net magnetic field or net magnetisation. This is often given the symbol M and at equilibrium it is aligned along the positive z axis (along B_o_) with the value, M_o_. It is often shown as an arrow or vector (Figure 2a).

The size of this net magnetisation is one of the key determinants of the maximum signal intensity that can be generated and used to form images. The greater the applied magnetic field strength, B_o_, the greater the excess of protons aligned with the magnetic field and the greater the size of the net magnetisation.

In order to generate a MR signal from the net magnetisation, the radiofrequency (rf) magnetic field described earlier is generated by the integral rf transmitter coil and used to deliver energy to the population of protons. This field is applied at a particular frequency, known as the Larmor frequency, ω_o _that is determined by the equation:

$$\omega_{\text{o}}=\gamma \;\times \;{\text{B}}_{\text{o}}$$

This equation is known as the Larmor equation. The constant γ is called the gyromagnetic ratio and has a value of 42.6 MHz/Tesla for the proton. The Larmor frequency is therefore proportional to the strength of the magnetic field and for 1.5 Tesla, the Larmor frequency is approximately 64 MHz. This is also known as the resonant frequency, as the protons only absorb energy (or resonate) at this characteristic frequency. The rf field is normally applied as a short pulse, known as an rf pulse.

### Radiofrequency pulses and flip angle

Before the rf pulse is switched on the net magnetisation, M_o_, is at equilibrium, aligned along the z-axis in the same direction as B_o _(Figure 2a). When the rf pulse is switched on, the net magnetisation begins to move away from its alignment with the B_o _field and rotate around it. The speed of this rotational motion, known as precession, is also at the Larmor frequency. The Larmor frequency is therefore also sometimes referred to as the frequency of precession. The movement of the net magnetisation away from alignment with B_o _is caused by a much slower rotation about the much smaller applied rf field, B_1_. This oscillating field, B_1 _is applied as a rotating field at right angles to B_o _in the plane of the x and y axes. As it rotates at the same frequency as the Larmor frequency, it appears as an additional static field to the rotating net magnetisation vector. The net magnetisation therefore rotates about both the B_o _and the B_1 _fields. As a result of these two rotations, the net magnetisation follows a spiral path from its alignment with the B_o _field (z-axis) towards a rotational motion in the plane of the x and y axes.

Remember that the net magnetisation is the result of the sum of many individual magnetic moments. So long as they rotate together (a condition known as coherence) they will produce a net magnetisation that is rotating. The greater the amount of energy applied by the rf pulse, the greater the angle that the net magnetisation makes with the B_o _field (the z axis). This depends upon both the amplitude and duration of the pulse. The rf pulse is switched off once the angle of precession has reached a prescribed value. This is known as the flip angle of the rf pulse (Figure 2b).

Once the rf pulse has caused the net magnetisation to make an angle with the z-axis, it can be split into two components (Figure 2b). One component is parallel to the z-axis. This is known as the z-component of the magnetisation, M_z_, also know as the longitudinal component. The other component lies at right angles to the z axis within the plane of the x and y axes and is known as the x-y component of the net magnetisation, M_xy_, or the transverse component. The transverse component rotates at the Larmor frequency within the xy plane and as it rotates, it generates its own small, oscillating magnetic field which is detected as an MR signal by the rf receiver coil. Radiofrequency pulses are commonly classified by both their flip angle and by their effect.

Radiofrequency pulses that generate an MR signal by delivering energy to the hydrogen spin population, causing the magnetisation to move away from its equilibrium position are known as excitation pulses. The 90° rf excitation pulse delivers just enough energy to rotate the net magnetisation through 90° (Figure 2c). This transfers all of the net magnetisation from the z-axis into the xy (transverse) plane, leaving no component of magnetisation along the z-axis immediately after the pulse. The system of protons is then said to be 'saturated' and the 90° rf pulse is therefore sometimes referred to as a saturation pulse. When applied once, a 90° rf pulse produces the largest possible transverse magnetisation and MR signal. This pulse is used to initially generate the signal for spin echo-based pulse sequences.

Low flip angle rf excitation pulses rotate the net magnetisation through a pre-defined angle of less than 90° (Figure 2b). A low flip is represented by the symbol α or can be assigned a specific value, e.g. 30°. Only a proportion of the net magnetisation is transferred from the z axis into the xy plane, with some remaining along the z axis. While a low flip angle rf pulse produces an intrinsically lower signal than the 90° excitation pulse described above, it can be repeated more rapidly as some of the magnetisation remains along the z-axis immediately after the pulse. This excitation pulse is used to generate the signal in gradient echo pulse sequences to control the amount of magnetisation that is transferred between the z-axis and the xy plane for fast imaging applications.

The 180° refocusing pulse is used in spin echo pulse sequences after the 90° excitation pulse, where the net magnetisation has already been transferred into the x-y plane. It flips the direction of the magnetisation in the x-y plane through 180° as it rotates at the Larmor frequency (Figure 2d). This pulse is used in spin echo-based techniques to reverse the loss of coherence caused by magnetic field inhomogeneities (described in the next section).

The 180° pulses are also used to prepare the net magnetisation before the application of an excitation pulse. These are known as inversion pulses and are used in inversion recovery or dark-blood pulse sequences. They are applied when the net magnetisation is at or close to equilibrium and invert the excess population of proton magnetic moments from being aligned to anti-aligned with the B_o _field (Figure 2e). Because the resultant magnetisation lies only along the z axis this pulse does not result in a detectable signal. It is used to prepare the z-magnetisation in inversion recovery pulse sequences and in black blood preparation schemes. This type of pulse is therefore also often referred to as a magnetisation preparation pulse.

### MR signal characteristics - T1, T2 and T2* relaxation

Immediately after the rf pulse the spin system starts to return back to its original state, at equilibrium. This process is known as relaxation. There are two distinct relaxation processes that relate to the two components of the Net Magnetisation, the longitudinal (z) and transverse (xy) components. The first relaxation process, longitudinal relaxation, commonly referred to as T1 relaxation is responsible for the recovery of the z component along the longitudinal (z) axis to its original value at equilibrium. The second relaxation process, transverse relaxation, is responsible for the decay of the xy component as it rotates about the z axis, causing a corresponding decay of the observed MR signal. Longitudinal and transverse relaxation both occur at the same time, however, transverse relaxation is typically a much faster process for human tissue. The signal decays away long before the spin system returns to its equilibrium state.

**Figure 3:**
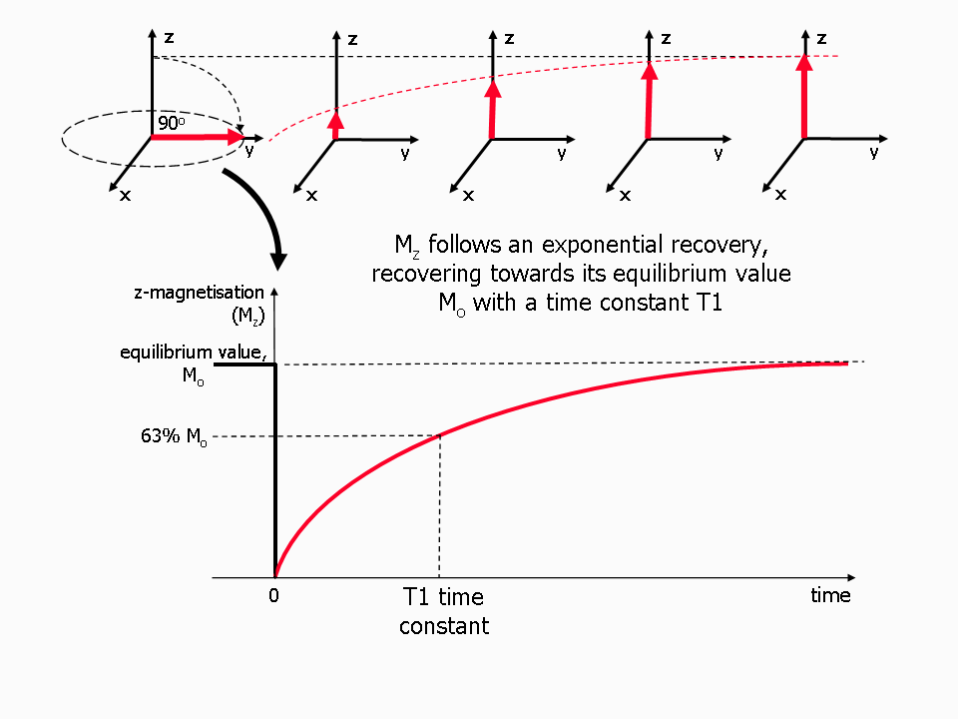
**T1 relaxation process**. Diagram showing the process of T1 relaxation after a 90° rf pulse is applied at equilibrium. The z component of the net magnetisation, M_z _is reduced to zero, but then recovers gradually back to its equilibrium value if no further rf pulses are applied. The recovery of M_z _is an exponential process with a time constant T1. This is the time at which the magnetization has recovered to 63% of its value at equilibrium.

T1 relaxation is an exponential process with a time constant T1. For example, if a 90° pulse (a saturation pulse) is applied at equilibrium, the z-magnetisation is saturated (reduced to zero) immediately after the pulse, but then recovers along the z-axis towards its equilibrium value, initially rapidly, slowing down as it approaches its equilibrium value (Figure 3). The shorter the T1 time constant is, the faster the relaxation process and the return to equilibrium. Recovery of the z-magnetisation after a 90° rf pulse is sometimes referred to as saturation recovery.

**Figure 4:**
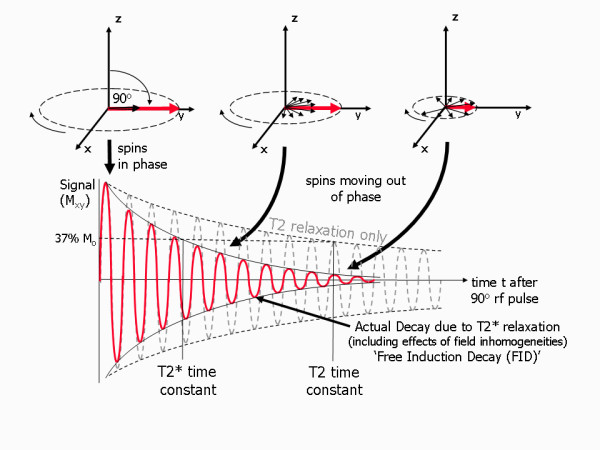
Transverse (T2 and T2*) relaxation processes. A diagram showing the process of transverse relaxation after a 90° rf pulse is applied at equilibrium. Initially the transverse magnetisation (red arrow) has a maximum amplitude as the population of proton magnetic moments (spins) rotate in phase. The amplitude of the net transverse magnetisation (and therefore the detected signal) decays as the proton magnetic moments move out of phase with one another (shown by the small black arrows). The resultant decaying signal is known as the Free Induction Decay (FID). The overall term for the observed loss of phase coherence (de-phasing) is T2* relaxation, which combines the effect of T2 relaxation and additional de-phasing caused by local variations (inhomogeneities) in the applied magnetic field. T2 relaxation is the result of spin-spin interactions and due to the random nature of molecular motion, this process is irreversible. T2* relaxation accounts for the more rapid decay of the FID signal, however the additional decay caused by field inhomogeneities can be reversed by the application of a 180° refocusing pulse. Both T2 and T2* are exponential processes with times constants T2 and T2* respectively. This is the time at which the magnetization has decayed to 37% of its initial value immediately after the 90° rf pulse.

Transverse relaxation can be understood by remembering that the net magnetisation is the result of the sum of the magnetic moments (spins) of a whole population of protons. Immediately after the rf pulse they rotate together in a coherent fashion, so that as they rotate they continuously point in the same direction as each other within the xy plane. The angle of the direction they point at any instant is known as the phase angle and the spins having similar phase angles are said at this initial stage to be 'in phase' (Figure 4). Over time, for reasons explained in a moment, the phase angles gradually spread out, there is a loss of coherence and the magnetic moments no longer rotate together and they are said to move 'out of phase'. The net sum of the magnetic moments is thus reduced, resulting in a reduction in the measured net (transverse) magnetisation. The signal that the receiver coil detects (if no further rf pulses or magnetic field gradients are applied) is therefore seen as an oscillating magnetic field that gradually decays (known as a Free Induction Decay or FID). There are two causes of this loss of coherence. Firstly, the presence of interactions between neighbouring protons causes a loss of phase coherence known as T2 relaxation.

This arises from the fact that the rate of precession for an individual proton depends on the magnetic field it experiences at a particular instant. While the applied magnetic field B_o _is constant, it is however possible for the magnetic moment of one proton to slightly modify the magnetic field experienced by a neighbouring proton. As the protons are constituents of atoms within molecules, they are moving rapidly and randomly and so such effects are transient and random. The net effect is for the Larmor frequency of the individual protons to fluctuate in a random fashion, leading to a loss of coherence across the population of protons. i.e. the spins gradually acquire different phase angles, pointing in different directions to one another and are said to move out of phase with one another (this is often referred to as de-phasing). The resultant decay of the transverse component of the magnetisation (M_xy_) has an exponential form with a time constant, T2, hence this contribution to transverse relaxation is known as T2 relaxation (Figure 4). As it is caused by interactions between neighbouring proton spins it is also sometimes known as spin-spin relaxation. Due to the random nature of the spin-spin interactions, the signal decay caused by T2 relaxation is irreversible.

The second cause for the loss of coherence (de-phasing) relates to local static variations (inhomogeneities) in the applied magnetic field, B_o _which are constant in time. If this field varies between different locations, then so does the Larmor frequency. Protons at different spatial locations will therefore rotate at different rates, causing further de-phasing so that the signal decays more rapidly. In this case, as the cause of the variation in Larmor frequency is fixed, the resultant de-phasing is potentially reversible. The combined effect of T2 relaxation and the effect of magnetic field non-uniformities is referred to as T2* relaxation and this determines the actual rate of decay observed when measuring an FID signal (Figure 4). T2* relaxation is also an exponential process with a time constant T2*.

### Significance of the T1 value

T1 relaxation involves the release of energy from the proton spin population as it returns to its equilibrium state. The rate of relaxation is related to the rate at which energy is released to the surrounding molecular structure. This in turn is related to the size of the molecule that contains the hydrogen nuclei and in particular the rate of molecular motion, known as the tumbling rate of the particular molecule. As molecules tumble or rotate they give rise to a fluctuating magnetic field which is experienced by protons in adjacent molecules. When this fluctuating magnetic field is close to the Larmor frequency, energy exchange is more favourable. For example, lipid molecules are of a size that gives rise to a tumbling rate which is close to the Larmor frequency and therefore extremely favourable for energy exchange. Fat therefore has one of the fastest relaxation rates of all body tissues and therefore the shortest T1 relaxation time. Larger molecules have much slower tumbling rates that are unfavourable for energy exchange, giving rise to long relaxation times. For free water, its smaller molecular size has a much faster molecular tumbling rate which is also unfavourable for energy exchange and therefore it has a long T1 relaxation time. The tumbling rates of water molecules that are adjacent to large macromolecules can however be slowed down towards the Larmor frequency shortening the T1 value. Water- based tissues with a high macromolecular content (e.g. muscle) therefore tend to have shorter T1 values. Conversely, when the water content is increased, for example by an inflammatory process, the T1 value also increases.

### Significance of the T2 value

T2 relaxation is related to the amount of spin-spin interaction that takes place. Free water contains small molecules that are relatively far apart and moving rapidly and therefore spin-spin interactions are less frequent and T2 relaxation is slow (leading to long T2 relaxation times). Water molecules bound to large molecules are slowed down and more likely in interact, leading to faster T2 relaxation and shorter T2 relaxation times. Water- based tissues with a high macromolecular content (e.g. muscle) tend to have shorter T2 values. Conversely, when the water content is increased, for example by an inflammatory process, the T2 value also increases. Lipid molecules are of an intermediate size and there are interactions between the hydrogen nuclei on the long carbon chains (an effect known as *J*-coupling) that cause a reduction of the T2 relaxation time constant to an intermediate value. Rapidly repeated rf pulses, such as those used in turbo or fast spin echo techniques, can have the effect of reducing *J*-coupling, resulting in an increased T2 relaxation time and higher signal intensity from fat [6].

### MR echoes

Whilst the FID can be detected as a MR signal, for MR imaging it is more common to generate and measure the MR signal in the form of an echo. This is because the magnetic field gradients that are used to localise and encode the MR signals in space cause additional de-phasing which disrupts the FID. The two most common types of echo used for MR imaging are gradient echoes and spin echoes. The following sections describe how these echoes are generated.

### Gradient echoes

**Figure 5:**
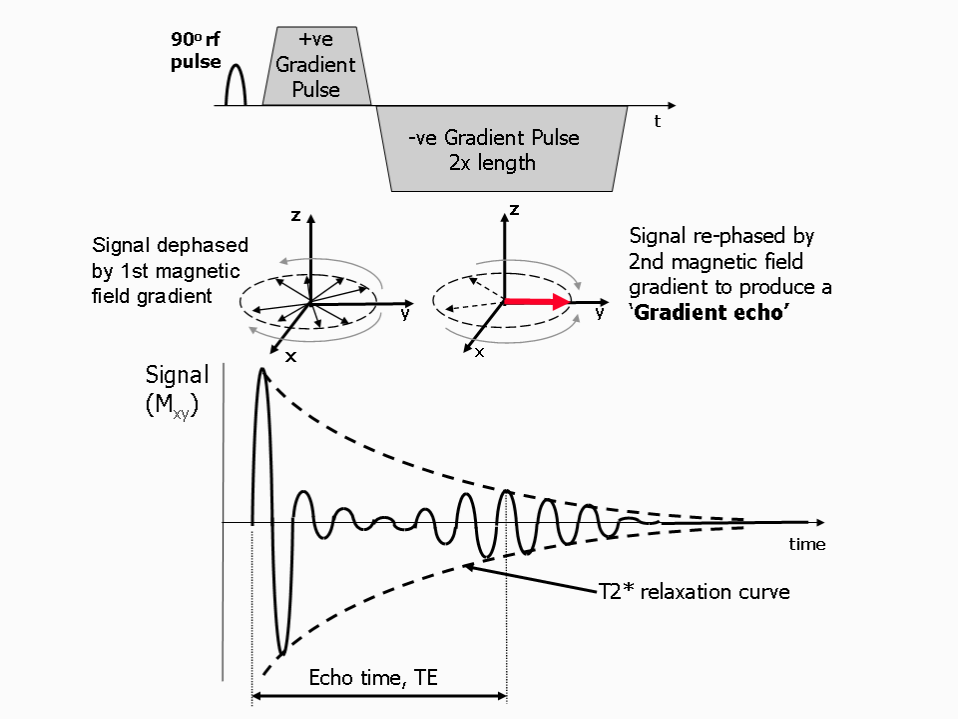
**Generating a gradient echo**. This diagram show how the reversal of a magnetic field gradient is used to generate a gradient echo. The application of the 1^st ^positive magnetic field gradient causes rapid de-phasing of the transverse magnetisation, M_xy_, and therefore the FID signal to zero amplitude. The application of the 2^nd ^negative magnetic field gradient reverses the de-phasing caused by the first gradient pulse, resulting in recovery of the FID signal to generate a gradient echo at the echo time, TE. Extension of the time duration of the second gradient to twice that of the first gradient causes the FID to then de-phase to zero. The maximum amplitude of the echo depends on both the T2* relaxation rate and the chosen TE.

Gradient echoes are generated by the controlled application of magnetic field gradients. Magnetic field gradients are used to produce a change in field strength and hence a corresponding change in Larmor frequency along a particular direction. When a magnetic field gradient is switched on it causes proton spins to lose coherence or de-phase rapidly along the direction of the gradient as they precess at different frequencies. This de-phasing causes the amplitude of the FID signal to rapidly drop to zero (Figure 5). The amount of de-phasing caused by one magnetic field gradient can however be reversed by applying a second magnetic field gradient along the same direction with a slope of equal amplitude but in the opposite direction. If the second gradient is applied for the same amount of time as the first gradient, the de-phasing caused by the first gradient is cancelled and the FID re-appears. It reaches a maximum amplitude at the point at which the spins de-phased by the first gradient have moved back into phase, or 're-phased'. If the second gradient then continues to be applied, the FID signal de-phases and disappears once more. The signal that is re-phased through the switching of the gradient direction is known as a gradient echo. The time from the point at which the transverse magnetisation (the FID) is generated by the rf pulse, to the point at which the gradient echo reaches it's maximum amplitude is known as the echo time (abbreviated TE). This can be controlled by varying the timing of the applied magnetic field gradients. If the echo time is chosen to be longer, more natural T2* de-phasing occurs and the maximum echo amplitude becomes smaller. In practice, the TE is set by the MR system operator (in milliseconds) as it determines, amongst other things, the influence of T2* on the image contrast.

### Spin echoes

**Figure 6:**
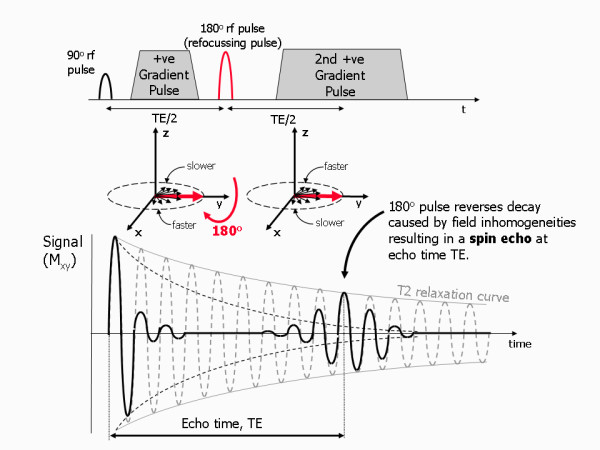
**Generating a spin echo**. The presence of magnetic field inhomogeneities causes additional de-phasing of the proton magnetic moments. The Larmor frequency is slower where the magnetic field is reduced and faster where the field is increased resulting in a loss or gain in relative phase respectively. After a period of half the echo time, TE/2, the application of a 180° rf pulse causes an instantaneous change in sign of the phase shifts by rotating the spins (in this example) about the y axis. As the differences in Larmor frequency remain unchanged, the proton magnetic moments the move back into phase over a similar time period, reversing the de-phasing effect of the magnetic field inhomogeneities to generate a spin echo. In addition to the effect of the 180° refocusing pulse, gradients are applied to de-phase and re-phase the signal for imaging purposes. Note that for spin echo pulse sequences, the second gradient has the same sign as the first, as the 180° pulse also changes the sign of the phase shifts caused by the first gradient.

Spin echoes are generated by the application of a 180° refocusing rf pulse after the 90° excitation pulse (Figure 6). While the de-phasing caused by T2 relaxation is a random, irreversible process, the additional de-phasing caused by the presence of static magnetic field inhomogeneities is potentially reversible. At a certain time after the initial generation of the FID signal, a proportion of the relative phase change for each proton spin is related to the local value of the applied magnetic field. The application of a 180° refocusing pulse rotates the spins through 180°, effectively changing the sign of the relative phase change within the xy plane. Where the previous relative phase change was positive due to a locally increased field, the 180° pulse causes it to become negative and visa versa. As the local field variations remain fixed, the spins still continue to have the same Larmor frequency, so a spin in an increased field continues to gain in phase, while a spin in a decrease field continues to lose phase. Because the sign of their phase shifts has been swapped halfway through by the 180° refocusing pulse, the spins all come back into phase causing the FID to increase in amplitude, reaching a maximum at the echo time, TE. For the spin de-phasing caused by the field non-uniformities to be completely reversed at time TE, the 180° pulse must be applied at time TE/2. The signal that re-appears (re-phases) through the application of the 180° rf refocusing pulse is known as a spin echo. After reaching a maximum amplitude at time TE, the signal again de-phases due to the T2* relaxation process. For the purposes of imaging, magnetic field gradients are also applied during the de-phasing period and during the measurement of the spin echo.

### Spin echo versus gradient echo

In general, because of the 180° refocusing pulse removes the de-phasing caused by magnetic field inhomogeneities, the amplitude of the spin echo signal is greater than the gradient echo signal. Imaging based on spin echo is also less affected by the presence of field inhomogeneities caused by metallic artefacts (e.g. sternal wires or metallic heart). Gradient echo imaging is however more affected by the presence of magnetic field inhomogeneities caused by iron and so is useful, for example, in the assessment of patients with increased iron deposition within the heart and liver.

## Localising and encoding MR signals to make an image

The MR echo signals produced above can be localised and encoded by applying magnetic field gradients as they are generated to produce an image. This is because the application of a magnetic field gradient causes the strength of the magnetic field and hence, the Larmor frequency to depend on position along that direction. The sections that follow describe the most commonly used method to build up a cross-sectional 2-dimensional image (or image slice) using a combination of rf pulses and gradient magnetic fields.

### Step 1 - Selection of an image slice

**Figure 7:**
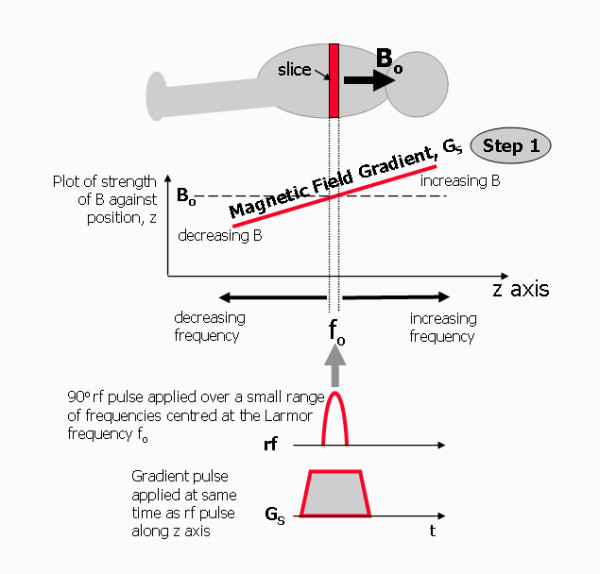
**Image formation, Step 1 - Selecting a slice**. For step 1 of image formation process, a slice of tissue is selected by applying a magnetic field gradient G_S _at the same time as the rf excitation pulse. The position along the gradient (in this example along the z axis) determines the Larmor frequency and resonance only occurs where this matches the frequency of the rf pulse, f_0_, defining a plane (slice) of tissue perpendicular to the z axis. In practice the rf pulse is applied over a small range of frequencies, thus defining the thickness of the slice.

First, the resonance of protons is confined to a slice of tissue. This is done by applying a gradient magnetic field at the same time as the rf excitation pulse is transmitted (Figure 7). The frequency of the rf pulse corresponds to the Larmor frequency at a chosen point along the direction of the applied gradient. The result is for resonance only to occur for protons in a plane that cuts through that point at right angles to the gradient direction, effectively defining a slice of tissue. This process is known as slice selection and the gradient is known as the slice selection gradient, G_S_. The orientation of the slice is determined by the direction of the applied gradient known as the slice selection direction (in the example of Figure 7 this is the z-direction). Rather than just a single frequency, the transmitted rf pulse is comprised of a small range of frequencies, known as the transmit bandwidth of the rf pulse. This gives the slice a thickness. The thickness of the slice is determined by the combination of the rf pulse bandwidth and the steepness (or strength) of the gradient.

### Step 2 - Phase encoding

**Figure 8:**
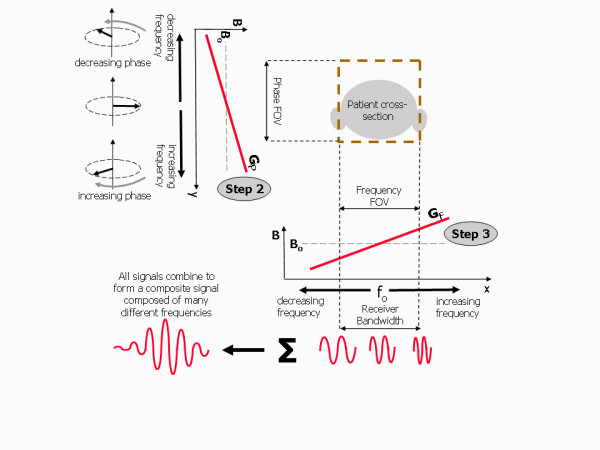
**Image formation, Steps 2 & 3 - Phase and frequency encoding**. For step 2 of the image formation process, a phase encoding gradient, G_P_, is applied in a direction along the selected image plane (in this case the phase encoding direction is along the y direction). This causes a range of phase shifts of the proton magnetic moments dependent on their position along the gradient as well as the slope and duration of the gradient. For step 3, following the phase encoding gradient, the frequency encoding gradient, G_F_, is applied also in the plane of the selected slice but perpendicular to the phase encoding direction. The MR signal echo is measured during this period. The frequency encoding gradient determines the Larmor frequency according to position along its direction (in this case, the x direction). The detected MR signal from the slice of tissue is therefore comprised of many different frequencies. The field of view is predefined and matched to a specific range of frequencies, referred to as the receiver bandwidth. See also Additional File 1.

Following slice selection, a phase encoding gradient, G_P_, is applied for a specified period (Figure 8). This causes the protons to rotate at different frequencies according to their relative position along the gradient. Where the gradient increases the magnetic field, the protons acquire a higher frequency of precession, while where the gradient decreases the magnetic field, the protons acquire a lower frequency of precession. The protons therefore also constantly change their relative phase according to their position along the gradient. When the gradient is switched off, the protons will have changed their relative phase by an amount depending on their position along the gradient. This process is known as phase encoding and the direction of the applied gradient is known as the phase encoding direction.

### Step 3 - Frequency encoding

Following the phase encoding gradient, the frequency encoding gradient, G_F_, is applied in a direction at right angles to it and in a similar way causes the protons to rotate at different frequencies according to their relative position along that direction gradient (Figure 8). This gradient is applied for longer, and at the same time the signal is measured or digitally sampled. The signal is comprised of a range of frequencies (or bandwidth), corresponding to the Larmor frequencies of the proton magnetic moments at their different locations along the gradient. This process is known as frequency encoding, the direction of the frequency encoding gradient defines the frequency encoding direction. The phase encoding and frequency encoding processes in steps 2 and 3 are further illustrated in an animation provided in Additional File 1.

**Figure 9:**
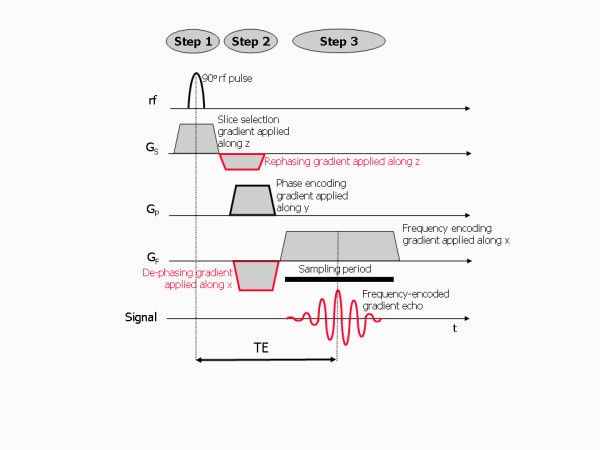
**Image formation - pulse sequence diagram**. A pulse sequence diagram showing the relative timing of the rf and gradient pulses applied as part of the three step process to localise and encode the MR signal for image formation. The frequency-encoded MR signal echo is measured during a sampling period centred at the echo time, TE. Additional gradient pulses (shown outlined in red) are required immediately after the slice selection gradient and immediately before the frequency encoding gradient. These additional pulses ensure that any de-phasing of the transverse magnetisation caused by the imaging gradients is cancelled once the echo time, TE, is reached. This results in the echo reaching its maximum possible signal at this point.

In summary, to localise the MR signal in three dimensions, three separate magnetic field gradients are applied in a three step process. For the examples in Figure 7 and 8 these gradients are applied in sequence with the slice-section gradient, G_S _applied along the z-axis, the phase-encoding gradient, G_P _applied along the y-axis and the frequency-encoding gradient, G_F _applied along the x- axis (Figure 9). This defines a slice perpendicular to the z axis i.e. a slice oriented in the transaxial plane. Other slice orientations are obtained by re-assigning each of the gradients to a different axis. An angled slice is obtained by combining gradients along two or more axes to perform each of the localisation tasks. The ability to define an arbitrary slice orientation is a key strength of magnetic resonance imaging, especially for cardiac applications, which necessitate double oblique angled slices to achieve standard views of the cardiac chambers and valve planes.

Note that in Figure 9 additional gradient pulses are shown both after the slice selection gradient and before the frequency encoding gradient. These extra gradient pulses are required to counteract de-phasing that is caused by these two imaging gradients, to ensure the maximum possible signal at the centre of the MR signal echo. The additional gradient pulses are applied along the same direction as the imaging gradients, but with opposite slope, so that the transverse magnetisation is brought back into phase. For the slice selection gradient, de-phasing only occurs during the second half of the slice selection gradient since the transverse magnetisation is only generated halfway through the applied rf pulse. It is therefore followed by a re-phasing gradient that is only half the length of the slice selection gradient. This ensures that de-phasing that occurs along the slice selection gradient is reversed. The frequency encoding gradient is normally preceded by a de-phasing gradient so that when the frequency encoding gradient is applied, the de-phasing is reversed by the first half of the frequency encoding gradient and the signal echo reaches its maximum amplitude at the centre of the sampling period.

### Image reconstruction

**Figure 10:**
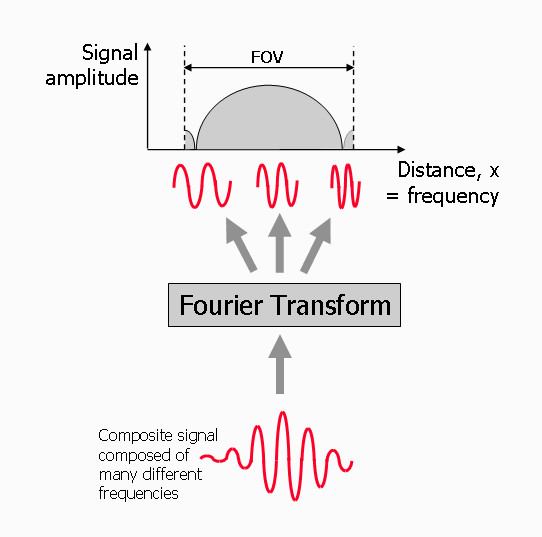
**Image reconstruction - frequency encoding**. The frequency encoded signal is analysed by a Fourier Transform to determine the contribution of each of the frequency components to each location along the frequency encoding gradient within the pre-defined field of view.

The frequency encoded signal is analysed using a Fourier transform. This is a mathematical tool that transforms the time-dependent MR signal into its different frequency components (Figure 10). The amplitude of each frequency component can be mapped onto a location along the frequency encoding gradient to determine the relative amount of signal at each location. The field of view in the frequency encoding direction is defined by the operator in mm or cm. The range of frequencies across this field of view is known as the receiver bandwidth and is determined by the amplitude (or slope) required for the frequency encoding gradient.

**Figure 11:**
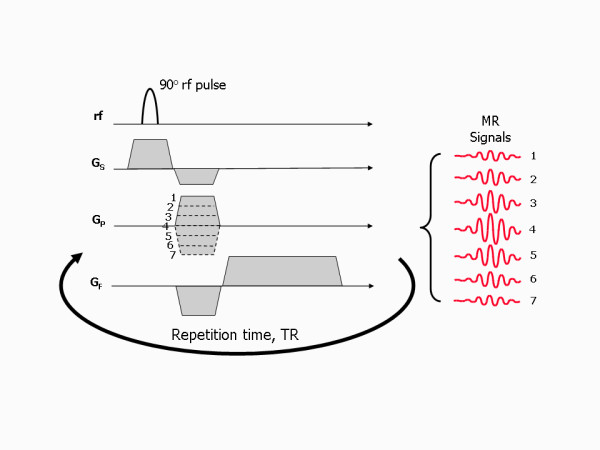
**Phase encoding steps and repetition time, TR**. To acquire sufficient information for image reconstruction, the pulse sequence is repeated a number of times, with an increment in the strength (or slope) of the phase encoding gradient being applied each time. In this example, 7 values of phase encoding gradient slope are used (shown by the dotted lines). Note that as the strength of the phase encoding gradient increases, this increases the amount of de-phasing along the gradient. When the strength (or slope) of the phase encoding gradient is zero (step 4), there is no de-phasing and the signal has its maximum possible amplitude. The time interval between each repetition is known as the repetition time, TR.

**Figure 12:**
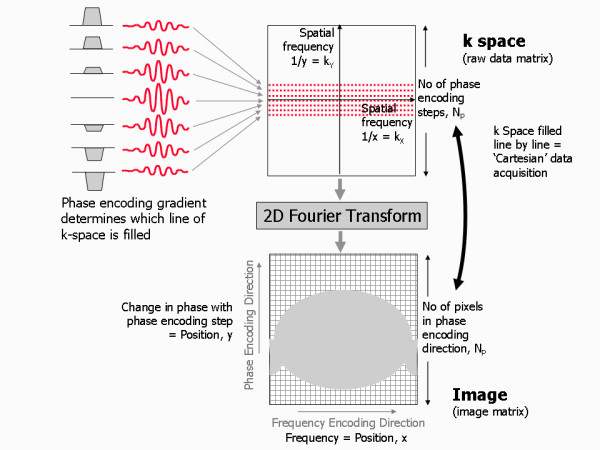
**Image reconstruction, k-space and image space**. The MR signals derived from each phase encoding step are stored in a raw data matrix, known as k-space. A two-dimensional Fourier transformation of this matrix results in the reconstruction of the image. The number of phase encoding steps determines the number of pixels in the image along the phase encoding direction. The coordinates of the image are the spatial coordinates x and y. The distribution of MR signal components in the image is determined by their frequency along the frequency encoding direction (in this case, x) and by their change in phase with each phase encoding step along the phase encoding direction (in this case, y). The Coordinates of k-space are the spatial frequencies k_x _= 1/x and k_y _= 1/y. The data points in k-space (the sampled MR signals) therefore represent the spatial frequencies content of the image. In a Cartesian data acquisition, the data points are stored line by line along the k_x _direction, with each line corresponding to a separately sampled MR signal. The position along k_x _depends on the time point during the sampling period. The location of each line of data points in the k_y _direction is determined by the amplitude and duration of the phase encoding direction at each phase encoding step.

While analysis of the encoded MR signal by the Fourier Transform provides the frequency content of the signal, the phase changes imparted by the phase encoding gradient cannot be decoded by a similar process. The Fourier Transform can only analyse a signal that changes over time. To enable this, a number of signal echoes are generated by repeating the above three-step process (slice selection, phase encoding and frequency encoding), each time applying the same slice selection and frequency encoding gradient, but a different amount of phase encoding (Figure 11). This is done by increasing the strength (or slope) of the phase encoding gradient for each repetition by equal increments or steps. For each phase encoding step the signal echo is measured, digitised and stored in a raw data matrix. The time interval between each repetition is known as the repetition time, TR. Once all the signals for a prescribed number of phase encoding steps have been acquired and stored, they are analysed together by a two-dimensional (2D) Fourier transform to decode both the frequency and the phase information (Figure 12).

### Repetition time and image acquisition time

The repetition time, TR is another important parameter that can be set by the operator in milliseconds. As we will see later it not only determines how fast MR images can be acquired but also affects the image contrast. The number of pixels in the phase encoding direction of the reconstructed image is determined by the number of phase encoding steps used, N_P_. The spatial resolution of the image therefore also depends on the number of phase encoding steps, and as a consequence is often limited by the image acquisition time, as

$$\text{Image acquisition time}=\text{TR}\times {\text{N}}_{\text{P}}$$

If a greater spatial resolution is required in the phase encoding direction (for a fixed field of view), the number of pixels in that direction (sometimes referred to as the acquired image matrix size) must be increased. This requires a greater number of repetitions, and therefore a longer image acquisition time.

### k-space

The way that the MR signals are generated and encoded by the use of magnetic field gradients gives rise to a particular relationship between the data points in the signal and those in the image. A single data point in an MR signal contributes a particular attribute to the whole image. Conversely, a single pixel in the image may have contributions from all of the MR signals collected. Just as each pixel occupies a unique location in image space, each point of an MR signal echo belongs to a particular location in a related space known as k-space [7]. There is an inverse relationship between the image space and k-space (Figure 12). Whereas the coordinates of the image represent spatial position (x and y), the coordinates of k-space represent 1/x and 1/y, sometimes referred to as spatial frequencies, k_x _and k_y_. The value of each point in k-space represents how much of a particular spatial frequency is contained within the corresponding image.

**Figure 13:**
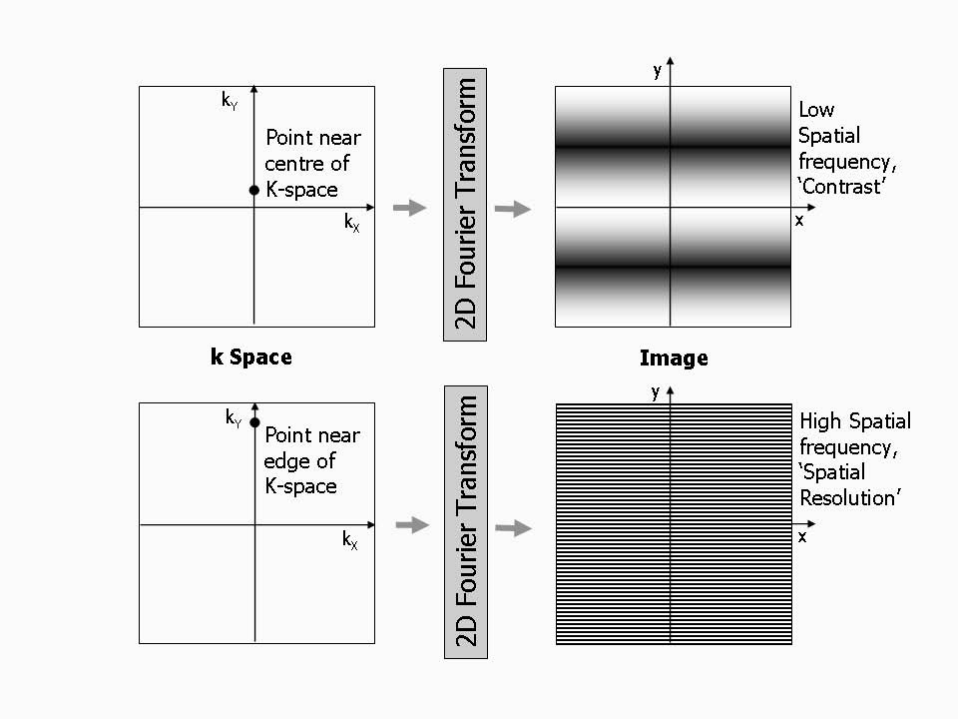
**k-space and spatial frequencies**. A single point in k-space defines a spatial frequency that can be represented as a wave in image space. A point close to the centre of k-space contributes a low spatial frequency, represented by a wave with broad peaks and troughs. This provides the signal content for large regions of uniform signal in the image and therefore the image contrast. A point at the edge of k-space contributes a high spatial frequency and is represented by a fine 'toothcomb' wave. The highest spatial frequency content defines the spatial resolution limit of the image.

**Figure 14:**
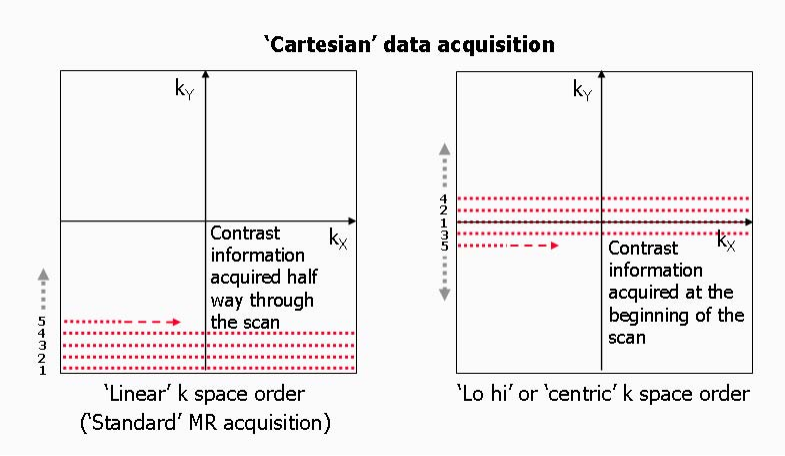
**k-space order**. Standard Cartesian MR data acquisition fills k-space by starting at one edge of k-space incrementing line by line until the opposite edge of k-space is reached. This is known as linear k-space order. 'Centric' or 'lo-hi' k-space order starts at the centre of k-space and fills lines of k-space outward to both edges in an alternating fashion.

A spatial frequency is difficult to picture. An image consisting of a single spatial frequency looks like a wave propagating across the image with bright and dark peaks and troughs (Figure 13). A low spatial frequency (arising from a point near the centre of k space) has peaks and troughs far apart and thus contributes mostly the signal content and contrast of the image. A high spatial frequency (arising from a point near the edge of k-space) has peaks and troughs close together and thus contributes fine detail or edges, effectively defining the spatial resolution of the image. To make an image that is a totally faithful representation of the imaged subject, it is important that the whole range of spatial frequencies is acquired (up to a maximum that defines the spatial resolution of the image), i.e. that the whole of k-space is covered. For standard imaging this is done by filling k-space with equally spaced parallel lines of signal data, line by line, along the k_x _direction. This is known as a Cartesian acquisition (Figure 12). The phase encoding gradient determines the position of the line being filled in the k_y _direction. Usually the amplitude of the phase encoding gradient is incremented in steps such that the next adjacent line in k-space is filled with each successive repetition, starting at one edge of k-space and finishing at the opposite edge. This is known as a linear phase encoding order (Figure 14). Choosing a different phase encoding step order is particularly important in some dynamic applications such as contrast enhanced angiography, where it is important to acquire the contrast information immediately at the start of the image data acquisition once the contrast agent reaches a particular vessel segment. In this case, the phase encoding gradient is incremented from zero, but with an alternating sign, starting at the centre of k space and working outwards to the edges of k-space, known as centric or low-high k-space order (Figure 14).

## Pulse sequences and image contrast

### Image contrast and weighting

One of the most important advantages of MR imaging over other imaging modalities is the ability to generate contrast between different soft tissue types. This is because different types of soft tissue have different characteristic T1 and T2 relaxation times. The dependence of the MR signal for a particular tissue on its relaxation properties is controlled by the choice of the pulse sequence parameters. For spin echo pulse sequences the excitation flip angle is fixed at 90° and the choice of TR and TE only control the influence of a tissue's T1 and T2 relaxation times on the signal. For gradient echo pulse sequences, the TR, TE and flip angle control the influence of a tissue's T1 and T2* relaxation times on the signal.

### Spin echo contrast and weighting

For spin echo pulse sequences the addition of a 180° refocusing pulse removes the effect of T2* relaxation and determines that the amplitude of the spin echo is influenced by T2 relaxation only. The TR and TE are chosen to weight the image contrast so that it is either primarily dependent upon the differences in T1 relaxation times (T1-weighted), or primarily dependent on the differences in T2 relaxation times (T2 weighted). If the parameters are chosen so that the image contrast is influenced by neither the T1 or T2 differences, the tissue signal is said to be primarily 'proton density' weighted. The TR controls the T1 weighting, while the TE controls the T2 weighting.

### T1-weighted spin echo

**Figure 15:**
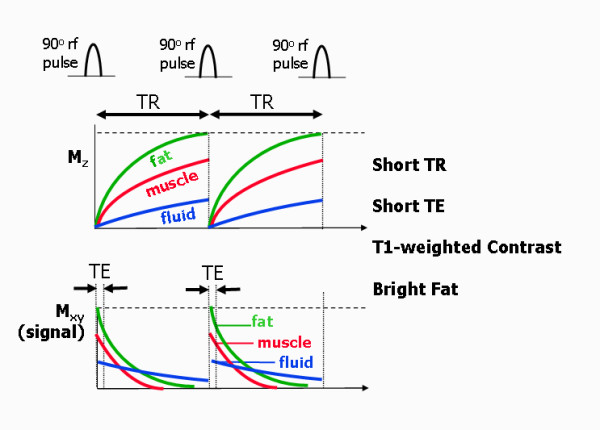
**T1-weighted spin echo**. T1 and T2 relaxation curves for a T1-weighted spin echo pulse sequence (180° pulse not shown) over two repetition periods and for three different tissues. The short TR leads to different amounts of recovery of the z-magnetisation for the three tissues, leading to T1-based contrast. The short TE minimises differences due to differences in T2 relaxation. T1-weighted images are characterised by bright fat and low signal intensity from static fluid.

The parameter choice for T1-weighted spin echo is a short TR and short TE (Figure 15). The choice of a short TR determines that tissues with a long T1 (e.g. fluid) will recover less than those with a short T1 (e.g. fat)). This determines the initial value of the transverse magnetisation, M_xy_, when the next rf pulse is applied. Tissues that have recovered less quickly will have a smaller longitudinal magnetisation before the next rf pulse, resulting in a smaller transverse magnetisation after the rf pulse. The short TE limits the influence of the different T2 decay rates. The resultant contrast is therefore said to be T1-weighted. T1 weighted spin echo images are typically characterised by bright fat signal and a low signal from fluid and are useful for anatomical imaging where high contrast is required between, fat, muscle and fluid. For cardiac imaging the pulse sequence is synchronised with the cardiac cycle and so the TR is determined by the patient's heart rate. For T1-weighted imaging the TR is set to one RR interval and the TE is set to a short value to minimise T2 weighting.

### T2-weighted spin echo

**Figure 16:**
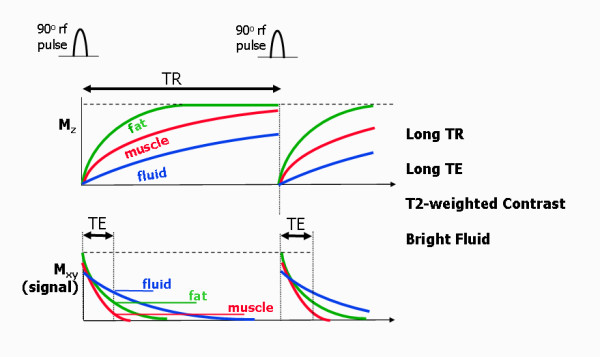
**T2-weighted spin echo**. T1 and T2 relaxation curves for a T2-weighted spin echo pulse sequence (180° pulse not shown) for three different tissues. The long TR minimises differences due to differences in T1 relaxation. The long TE leads to different amounts of decay of the xy-magnetisation for the three tissues, leading to T2-based contrast. T2-weighted images are characterised by bright fluid and low signal intensity from muscle.

The parameter choice for T2-weighted spin echo is a long TR and long TE (Figure 16). The choice of a long TR allows the z-magnetisation to recover close to the equilibrium values for most of the tissues, therefore reducing the influence of differences in T1 relaxation time. The longer echo time however allows more decay of the xy component of the magnetisation. The differential rate of decay between a tissue with a short T2 (e.g. muscle) and a tissue with a long T2 (e.g. fluid), leads to a difference in signal that is said to be T2-weighted. The short T2 leads to a reduced signal intensity, while the long T2 leads to an increased signal intensity. These images are characterised by bright fluid and are useful for the depiction of fluid collections and the characterisation of cardiac masses and oedema. For T2-weighted imaging with cardiac synchronisation, the TR is set to two or three RR intervals, depending on the heart rate, to provide a long TR and minimise T1-weighting.

### Proton density-weighted spin echo

**Figure 17:**
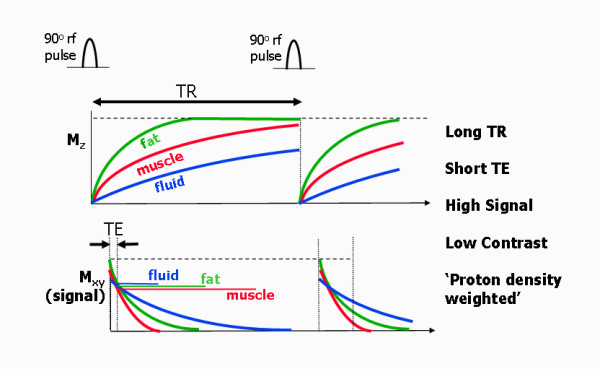
**'Proton density'-weighted spin echo**. T1 and T2 relaxation curves for a 'proton density'-weighted spin echo pulse sequence (180° pulse not shown) for three different tissues. The long TR minimises differences due to differences in T1 relaxation, while the short TE minimises differences due to differences in T2 relaxation, leading to T2-based contrast. 'Proton density'-weighted images are characterised by high signal intensity from most tissues and low tissue contrast.

The parameter choice for proton density-weighted spin echo is a short TR and short TE (Figure 17). The choice of long TR allows recovery of the z-magnetisation for most tissues, therefore reducing the influence of differences in T1 relaxation time and the 90° excitation pulse therefore transfers a similar amount of signal into the xy plane for all tissues. The choice of a short TE limits the amount of T2 decay for any tissue at the time of measurement. This results in a high signal from all tissues, with little difference between them. So the signal amplitude is not particularly affected by the T1 relaxation properties, or by the T2 relaxation properties. The primary determinant of the signal amplitude is therefore the equilibrium magnetisation of the tissue and the image contrast is said to be 'proton density'-weighted. This type of weighting is useful where the depiction of anatomical structure is required, without the need to introduce soft tissue contrast.

### Black blood contrast of spin echo pulse sequences

**Figure 18:**
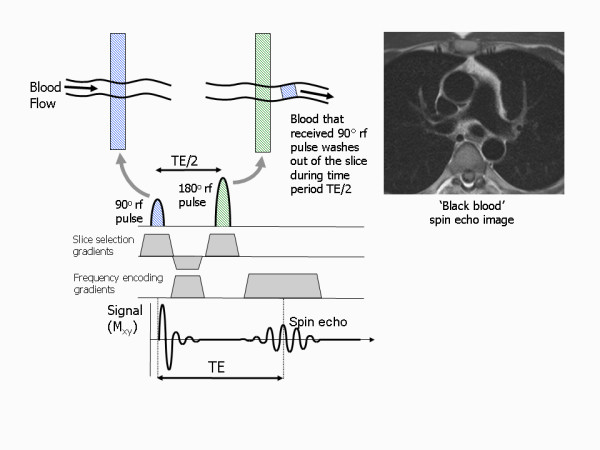
**Black blood contrast from spin echo pulse sequences**. Black blood appearance using spin echo pulse sequences is caused by the motion of blood through the image slice between the 90° and 180° pulses. The 90° pulse causes resonance in all the tissue within the slice, however a spin echo signal is only produced when the same tissue and blood also receive the 180° refocusing pulse. Blood that moves out of the slice during the time between the 90° and 180° pulses doe not produce a spin echo, resulting in a signal void as seen in the major vessels of the black blood spin echo image example.

The spin echo pulse sequence generates images that have intrinsic black blood contrast when imaging fast moving blood flowing through the image slice [8]. This is because it uses two pulses, the 90° and 180° pulses, to produce the spin echo signal. Both of these pulses are slice-selective but are separated by a time equal to half the echo time (Figure 18). The transverse magnetisation of blood flowing through the slice that moves out of the slice between the two pulses is not refocused by the 180° pulse and does not contribute to the generation of a spin echo. If the flow is sufficiently rapid for all the blood receiving the 90° pulse to move out of the slice, this results in a signal void. This effect is also known as the spin washout effect, which describes the 'washout' of proton spins form the image slice that would otherwise be refocused and contribute to the spin echo signal. When there is significant blood flow *through *the slice, this 'black blood' appearance provides high intrinsic contrast between the blood pool and the heart and blood vessel walls, which, in the early days of cardiac MR, made spin echo the pulse sequence of choice for anatomical imaging. The spin washout effect is reduced, however, where blood moves either slowly through or within the plane of the image slice. This results in a loss of dark blood contrast and a high residual blood signal that can lead to significant ghosting artefacts. The spin echo-based approach has therefore been modified for widespread clinical use by the addition of a black blood magnetisation preparation scheme [9] which provides more reliable black blood contrast (see later).

### Gradient echo contrast and weighting

There are a number of types of gradient echo pulse sequence, each having quite different contrast behaviour [10]. The two main types of gradient echo pulse sequence used for cardiac cine imaging have the generic names, spoiled gradient echo and balanced steady state free precession (bSSFP). MR manufacturers also have their own names for these pulse sequences and these are also given in the following sections:

### Spoiled gradient echo

Siemens:   FLASH   Fast Low Angle Shot

Philips:   T1 FFE   T1-weighted Fast Field Echo

GE:   SPGR   Spoiled GRASS (Gradient Recalled

Acquisition in the Steady State)

Gradient echo pulse sequences in cardiac imaging typically use very short TR values (<10 milliseconds) which gives rise to a more complex contrast behaviour. The TR values used are much shorter than the T2 relaxation times of blood and myocardium. This means that unless the transverse magnetisation generated by each rf pulse is destroyed after it has been sampled, it would still exist when the next rf pulse is applied. This can potentially contribute to, or interfere with, the signal during the following TR. In spoiled gradient echo, this signal is de-phased (or spoiled) either using a spoiler gradient at the end of each TR period, or by using a technique known as rf spoiling [11] so that its contribution to subsequent TR periods is suppressed.

**Figure 19:**
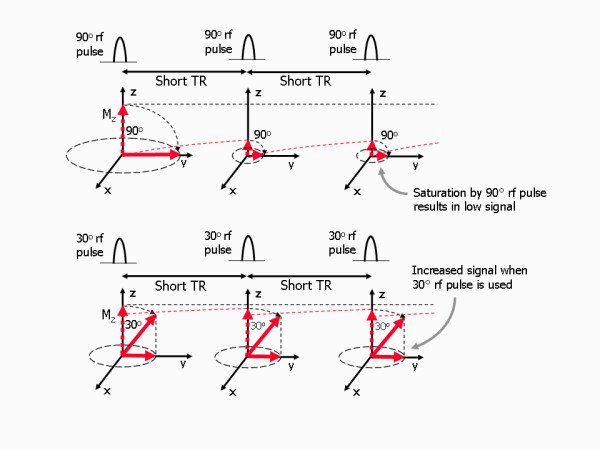
**Use of low flip angles with gradient echo pulse sequences**. Gradient echo sequences can use a variable (low) flip angle for the excitation pulse which allows much shorter TR values to be used without losing too much signal. When a 90° rf pulse is used (top row), the short TR allows very little recovery between rf pulses. The z-magnetisation quickly reduces, resulting in a low signal amplitude when it is transferred into the xy plane. The use of a low flip angle (in this case, 30°, bottom row), allows the z-magnetisation to remain much closer to its equilibrium value. This, when transferred into the xy plane, results in a much larger signal in comparison.

Spoiled gradient echo pulse sequences follow a similar contrast behaviour to that described for spin echo, however there are some key differences. For gradient echo pulse sequences the absence of a 180° refocusing pulse determines that the amplitude of the gradient echo at the TE, is influenced by T2* relaxation. Furthermore, a variable flip angle for the excitation pulse, as well as the TR and TE, is used to control image contrast. These three parameters can be chosen to weight the image contrast so that it is either primarily dependent upon the differences in T1 relaxation times (T1-weighted), or primarily dependent on the differences in T2* relaxation times (T2*-weighted). The use of a low flip angle is important for spoiled gradient echo techniques [12,13] as it allows the TR to be reduced to much lower values than are possible for spin echo techniques (Figure 19). The low flip angle, α, is normally chosen to be less than 90° (typically 30° or less). While this initially results in a smaller transverse magnetisation, (and therefore signal) as only a proportion of the z-magnetisation is transferred into the xy plane, the magnetisation that remains along the z axis returns back to its equilibrium value sooner, allowing the repetition time to be reduced. In this case, a much larger transverse magnetisation is achieved following the subsequent low flip angle pulses, compared to that generated by a train of 90° pulses in combination with the same very short TR. This is known as low flip angle imaging and it forms the main basis by which spoiled gradient echo pulse sequences are used for fast imaging.

### T1-weighted spoiled gradient echo

For spoiled gradient echo, T1-contrast is controlled by both the TR and the flip angle.

Cardiac cine imaging requires very short repetition times to be used and so resultant spoiled gradient echo sequence, with both a short TR(<10 ms) and TE (<5 ms), combined with a flip angle of around 30° essentially behaves as a T1-weighted pulse sequence. As a very short TR is used, myocardial tissue or blood that remains in the slice becomes saturated. This sequence thus relies on the flow of blood to generate contrast.

### T2*-weighted spoiled gradient echo

T2* weighting with spoiled gradient echo pulse sequences is achieved by increasing the TR and TE to relatively long values. As the T2* values for tissues are shorter than the T2 values, the echo times chosen to achieve T2* weighting with gradient echo are also much shorter than the echo times required to achieve T2 weighting with spin echo sequences. For T2*-weighted gradient echo, the image contrast is strongly influenced by the presence of magnetic susceptibility effects and can be used to detect the presence of iron, for example where there is haemorrhage or iron loading of tissue [14].

### Balanced steady state free precession (bSSFP)

GE:   FIESTA   Fast Imaging Employing Steady sTate Acquisition

Philips:   bFFE   balanced Fast Field Echo

Siemens:   True FISP   True Fast Imaging with Steady Precession

Balanced SSFP gradient echo sequences are designed to ensure that the transverse magnetisation is not spoiled but brought back into phase at the end of each TR period when the next rf pulse is applied. This then carries over into the next repetition and is superimposed onto the to the transverse magnetisation generated by that rf pulse. After a number of repetitions this gives rise to a steady state condition where the transverse magnetisation from two or three successive repetition periods combine to give a much greater signal [15,16].

The contrast behaviour of bSSFP sequences is very different to that of the spoiled gradient echo sequences. SSFP contrast is related to the tissue's T2/T1 ratio, with fluid and fat in particular appearing as brighter than other tissues. Because the transverse magnetisation originating from several TR's are combined, the MR signal amplitude for bSSFP is much greater compared to spoiled gradient echo. The increased signal allows a higher receiver bandwidths to be used, resulting in a shorter TE and TR compared to spoiled gradient echo pulse sequences and therefore improved imaging efficiency. However, if the magnetic field is not uniform, the transverse magnetisation from different TRs can destructively cancel rather than add together in areas of magnetic field inhomogeneity, making the SSFP technique prone to dark banding artefacts across the image [16]. It is therefore very important to ensure that the magnetic field is as uniform as possible over the region of interest to achieve images that are free of banding artefacts. This is achieved by a patient-specific process called dynamic shimming which uses the magnetic field gradients to correct for patient induced field inhomogeneities. Keeping the TR as short as possible also helps to minimise the banding artefacts observed in bSSFP imaging.

### Bright blood contrast of gradient echo pulse sequences

In contrast to the spin echo sequence, the gradient echo sequence only uses one rf pulse to generate the signal and so the spin washout effect does not apply and the signal from flowing blood is usually visible. Indeed, rather than suffering from a reduction in signal, flowing blood often appears with an apparently increased signal, compared to the surrounding tissues [17]. The gradient echo pulse sequence is therefore commonly referred to as a bright blood imaging technique.

**Figure 20:**
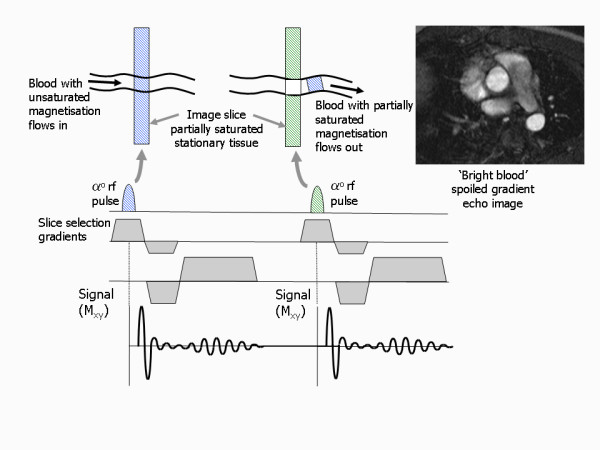
**Bright blood contrast from gradient echo pulse sequences**. Gradient echo pulse sequences often use very short repetition times to enable fast imaging. This results in limited recovery of the tissue magnetisation between pulses. Tissue that remains within the slice therefore has a reduced signal. Blood that flows through the image slice is constantly being replaced by fully magnetised blood which is able to generate a much higher signal when the excitation pulse is applied, resulting in a bright blood appearance. Flow-related enhancement can clearly be seen in the ascending and descending aorta on the bright blood spoiled gradient echo image example. Note that there is less flow-related enhancement within the main pulmonary artery and atria as the blood is not flowing through the image slice and is therefore partially saturated.

The very short TR (TR < 10 ms) employed for the purpose of fast imaging means that the magnetisation of tissue that remains in the image slice becomes partially saturated as rf pulses are rapidly applied to the same tissue, as there is little time for recovery of the z-magnetisation between pulses (Figure 20). This has the effect of reducing the signal from stationary tissue or blood that remains within the slice. Flowing blood that moves into the slice, however, has not received any previous pulses and the spin population is therefore fully magnetised. The moving blood is therefore able to generate a much higher signal than the surrounding tissue, thus the blood signal appears enhanced or bright. This effect is known as inflow enhancement and is particularly important for spoiled gradient echo pulse sequences for which saturation of the blood signal plays a greater part. When there is significant blood flow through the slice, the bright blood signal provides good intrinsic contrast between the blood pool and the heart and blood vessel walls. The flow enhancement effect in spoiled gradient echo techniques is also used as the basis for time-of-flight MR angiography (TOF MRA). Where the blood is flowing slowly through the slice or in a direction within the plane of the image slice, inflow enhancement is reduced and the bright blood contrast is reduced. Inflow enhancement plays less of a role in bSSFP pulse sequences as the bright blood signal mainly arises because of the intrinsic contrast based on the higher T2/T1 ratio for blood compared to that of the myocardium and vessel walls.

### Spin echo vs. gradient echo

The 90° excitation pulse used by the spin echo pulse sequence transfers all of the available z magnetisation into the transverse plane. This combined with the 180° refocusing pulse gives the largest possible signal provided the magnetisation is allowed to recover sufficiently between repetitions. These two attributes make the spin echo technique ideally suited when the primary goal is to achieve images with a high signal-to-noise ratio and a reduced sensitivity to artefacts caused by magnetic field inhomogeneities. Fast gradient echo pulse sequences are used where imaging speed is more important than image quality. The absence of the 180° refocusing pulse in the gradient echo sequence leads to signal loss in the presence of magnetic susceptibility effects and at the boundaries between water and fat-based tissues. Flowing blood also appears differently between the two sequences, with spin echo giving an intrinsic black blood appearance and gradient echo pulse sequences giving an intrinsic bright blood appearance. The key differences between gradient echo and spin based techniques are summarised in Table 1.

**Table 1:** Summary of key differences between gradient echo and spin echo sequences

	Spin echo	Gradient echo (spoiled)	Gradient echo (bSSFP)
Flip angle (excitation pulse)	90°	variable 5°-40°	variable 50°-70°

180° refocusing pulse	Yes	No	No

Contrast weighting	T1, T2, 'proton density'	T1, T2*	T2/T1 ratio

Short Repetition time (T1- weighting)	400-800 ms	3-400 ms (depends on flip angle)	3-5 ms

Short echo time (minimise T2 or T2* weighting)	6-25 ms	1-3 ms	1-3 ms

Long echo time (for T2 or T2* weighting)	60-100 ms	7-15 ms	N/A

Long Repetition time (minimises T1 weighting)	1500-2500 ms	100 ms - (depends on flip angle)	N/A

Shortest practical TR	200 ms	2-5 ms	2-5 ms

Intra-voxel signal loss (susceptibility, iron)	No	Yes	Yes

Signal from blood flowing through the slice	Dark (spin washout)	Bright (inflow enhancement)	Bright (intrinsic T2/T1 contrast)

Cardiac Applications	T1, T2 and 'proton density'-weighted anatomical imaging and tissue characterisation	Fast cine imaging, Contrast-enhanced MR angiography, flow imaging, T2*-weighted imaging for iron loading.	Fast cine imaging

## Cardiovascular MR

### Synchronising with the cardiac cycle

**Figure 21:**
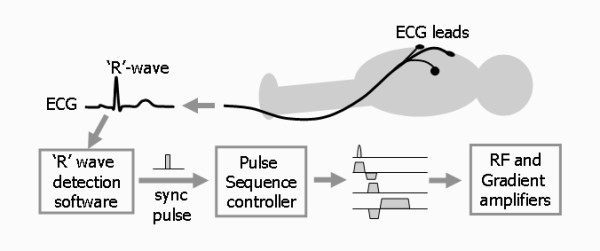
**ECG synchronisation of imaging pulse sequences**. Cardiac synchronisation is achieved by obtaining an ECG signal from the patient using MR-compatible ECG electrodes and leads. A software algorithm is then used to detect the QRS complex and generate a synchronisation pulse. This initiates the pulse sequence controller to produce rf and gradient pulse waveforms that are amplified to drive the rf transmitter and gradient coils. This is then repeated, with each cardiac cycle triggering a new repetition of the pulse sequence.

To capture an image of the heart that is unaffected by motion requires an image to be acquired in just a few tens of milliseconds. This means both limiting the number of phase encoding steps (and thus the spatial resolution) and making the TR as short as possible. Whilst this can be done, it is at the cost of accepting a significant reduction in image quality. On the other hand, to achieve acceptable image quality, the image acquisition time becomes too long to 'freeze' heart motion. For routine CMR therefore, the MR signals are acquired over multiple heart beats, synchronising the pulse sequence and therefore the signal acquisition to a particular time point in the cardiac cycle. Cardiac synchronisation is achieved by using the patient's ECG signal, obtained by applying ECG pads and leads onto the patient's chest (Figure 21). Software is used to detect the 'R' wave of the ECG and to generate a synchronisation pulse which is then used to synchronise the MR data acquisition [18]. This enables images of the beating heart to be obtained either at a single time point (still imaging) or at multiple time points through the cardiac cycle (cine imaging).

### Dealing with respiratory motion

For conventional spin echo and gradient echo imaging techniques, the phase encoding gradient is incremented with each successive heart beat, acquiring a single line of k-space each heart beat and resulting in imaging times of several minutes. This means that images using these techniques are degraded by respiratory motion. Image degradation caused by respiratory motion can be reduced by using one of three possible approaches, namely respiratory compensation methods (respiratory gating), cardiac synchronised fast imaging techniques combined with patient breath-holding or ultra-fast (single-shot) imaging techniques (the so called real-time imaging techniques described later). In practice, most cardiac imaging is performed with patient breath-holding combined with fast imaging techniques and these are described in the following section.

### Fast imaging techniques

Conventional imaging techniques acquire only one phase encoding step (one line of k-space) per heart beat. Thus the TR for those pulse sequences is defined by the patient's heart rate and is equal to the R-R interval. It therefore invariably takes several minutes to acquire an anatomical image dataset with conventional spin echo (SE) or a cine image dataset with conventional gradient echo sequences (spoiled gradient echo or bSSFP pulse sequences). In order to overcome this limitation to achieve shorter image acquisition times, fast imaging techniques acquire more than one line of k-space in each heart beat [19]. This fills up k-space more rapidly, leading to shorter image acquisition times. Spin echo and gradient echo pulse sequences that use this principle are known as turbo or fast pulse sequences.

### Still Imaging (Black blood anatomical imaging)

The ECG synchronisation technique used for still imaging is known as triggering. The synchronisation pulse is used as a trigger to initiate the pulse sequence at a particular time point after the R-wave in each cardiac cycle. This time point is known as the trigger delay and is selectable by the system operator to determine the point in the cardiac cycle at which the heart is to be imaged. This still imaging approach can be used for myocardial viability assessment or coronary angiography anatomical imaging, but the most routine application is to use it in combination with a fast or turbo spin echo pulse sequence to acquire black blood images for anatomical imaging.

### Turbo (or fast) spin echo

Philips, Siemens   TSE   Turbo Spin Echo

GE   FSE   Fast Spin Echo

**Figure 22:**
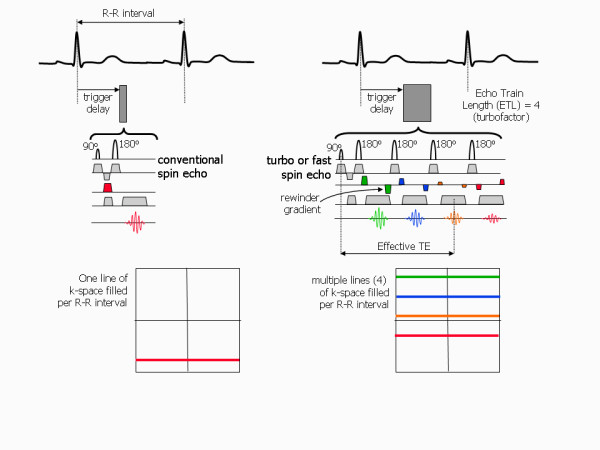
**Accelerated spin echo imaging with turbo or fast spin echo**. The conventional spin echo pulse sequence (left) applies a single 180° pulse following the 90° pulse to generate a single spin echo, filling only one line of k-space each R-R interval. The turbo or fast spin echo pulse sequence (right) applies multiple 180° pulses following the 90° pulse to generate multiple spin echoes. Multiple lines of k-space are filled within each R-R interval by applying a phase encoding gradient with a different amplitude to each echo. In this diagram each phase encoding gradient is colour coded to identify which corresponding line of k-space is filled. The phase encoding applied to each echo is removed by applying an equal and opposite re-winder gradients after each echo is sampled. In this example, four 180° pulses are applied to generate four spin echoes (echo train length or turbofactor = 4). This provides a four-fold acceleration in scan time.

The conventional spin echo (SE) pulse sequence generates a single spin echo signal by the use of an excitation pulse followed by a 180° refocusing pulse. The turbo or fast spin echo pulse sequence [20,21] generates multiple echoes by applying multiple 180° pulses after the initial 90° pulse (Figure 22). Each time a spin echo becomes de-phased due to the presence of magnetic field inhomogeneities, the de-phasing is reversed by the application of a further 180° pulse, generating a further corresponding spin echo. Each echo is used to fill a new line of k-space by applying a different amount of phase encoding to each echo, prior to data sampling. The number of echoes acquired for each excitation pulse is known as the echo train length (ETL) or 'turbofactor'. This effectively defines the factor by which the pulse sequence is accelerated. For example, if an ETL of 2 is chosen, four lines of k-space are filled within each TR rather than one. Typically echo train lengths of 15 or 16 are used in order to reduce the imaging time to within a breath-hold period. Note that each successive echo in the echo train has a different echo time, with the amplitude of each echo diminishing as it's echo time increases according to T2 decay. The effective echo time is defined as that of the echo which is acquired closest to the centre of k-space (with the smallest phase encoding gradient) as this is the echo that has the greatest influence on the image contrast. In comparison to conventional spin echo imaging, images acquired using turbo or fast spin echo pulse sequences are characterised by high signal intensity from fat, despite its intermediate T2 value [6]. This increase in signal is attributed to the application of the rapidly repeated 180° rf pulses that breaks down an interaction known as *J*-coupling that is present between the hydrogen nuclei in molecules with long carbon chains. This interaction has the effect of reducing the T2-relaxation time and therefore the signal intensity in conventional spin echo images. For cardiac imaging, turbo or fast spin echo pulse sequences are commonly used in combination with a double inversion 'black-blood' magnetisation preparation scheme to acquire anatomical images of the heart and major vessels.

### Black Blood Double Inversion preparation pulses

**Figure 23:**
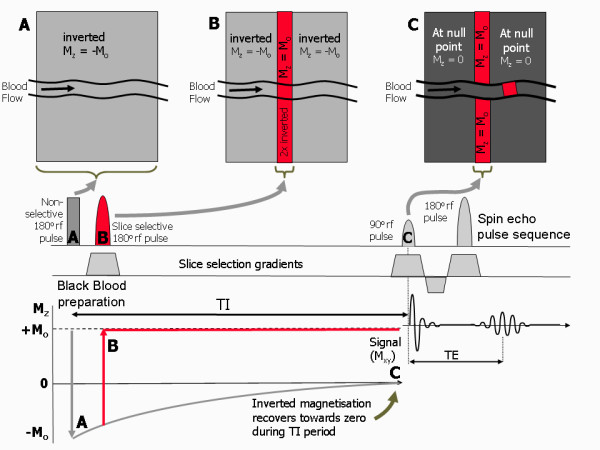
**Double inversion black-blood preparation scheme**. The double-inversion black-blood preparation pulse scheme uses two 180° inversion rf pulses to make the suppression of the blood signal more effective. The first inversion pulse, A, is not slice selective and inverts the magnetisation of all the tissue within range of the rf transmitter coil. The second inversion pulse, B, is a slice selective pulse that restores the magnetisation of the tissue within the intended image slice. The net effect of pulses A and B is to invert the magnetisation of all the tissue outside the intended image slice (shown in grey). After a prescribed inversion recovery period, TI, chosen as the time taken for the blood magnetisation to reach zero, an excitation pulse, C, is applied to generate a signal that is dependent on the current value the z-magnetisation of tissue and blood within the slice. During that same period, the non-inverted blood within the slice (red) is likely to have been replaced by the blood from outside the slice that has been inverted (grey) resulting in a signal void within the vessel.

Reliance on the spin washout effect alone to produce dark blood contrast often leads to inconsistent results due to insufficient blood flow. In order to improve the effectiveness of black blood imaging it is common to use a black blood magnetisation preparation scheme in combination with the spin echo pulse sequence [9,22]. The preparation scheme consists of the addition of two 180° rf inversion pulses followed by a delay, prior to the spin echo pulse sequence (Figure 23). The first 180° pulse inverts the magnetisation of all blood and tissues within range of the rf body transmitter coil. The second 180° pulse re-inverts the magnetisation only within the slice of tissue to be imaged. The net effect of these two pulses is to invert the magnetisation of blood and tissue outside the slice, while the magnetisation within the slice remains close to its equilibrium value. There is then a time delay before the excitation pulse (Time from Inversion, TI). During this time, the inverted blood magnetisation recovers due to T1 relaxation from its initial negative value, towards its positive equilibrium value. The TI is calculated to be equal to the time it takes the inverted magnetisation of blood to pass through zero. At that time, the 90° excitation pulse of the turbo or fast spin echo pulse sequence is applied. During the same TI period, blood flow causes the blood with inverted magnetisation to move into the image slice, replacing the blood that has remained at equilibrium. As the spin echo pulse sequence is applied at the same time as the inverted blood magnetisation reaches zero, no signal is produced from the blood. The double inversion pulse black blood preparation scheme provides much better signal suppression as the time delay used here (TI) is much greater than the time period (TE/2) that gives rise to the intrinsic black blood contrast of the conventional spin echo pulse sequence.

### Anatomical imaging with black blood FSE/TSE pulse sequences

**Figure 24:**
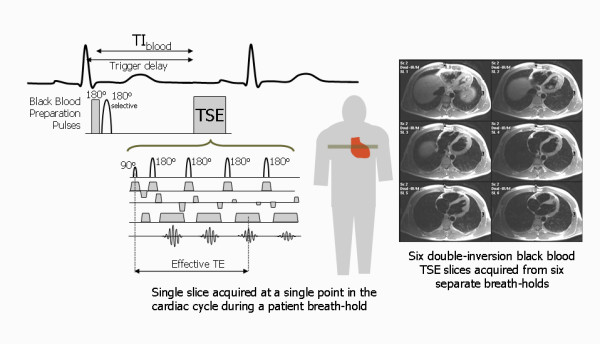
**Black-blood anatomical imaging with turbo or fast spin echo**. The Black Blood Preparation scheme is applied at the beginning of the cardiac cycle with the image acquisition in diastole. The exact duration of the Black Blood inversion, TI_Blood _is calculated to provide the best blood suppression and depends on the heart rate and the number of heart beats between each trigger pulse (typically TI_Blood _= 400-600 milliseconds). The turbo or fast spin echo pulse sequence enables one or two slices to be acquired within a single patient breath-hold. The six images to the right have been acquired using six separate breath-holds.

The most commonly-used pulse sequence for anatomical imaging combines the black-blood preparation scheme with the turbo or fast spin echo pulse sequence (Figure 24). The black-blood preparation scheme provides consistently high contrast between the heart and vessel walls and the blood pool. The use of the turbo or fast spin echo pulse sequence with an echo train length (turbofactor) of between 15-20 shortens the image acquisition time so that it falls within a typical breath-hold period. One or two slices are typically acquired within each breath-hold period. Adjustment of the k-space order within the echo train controls the effective echo time and therefore the T2-weighting of the contrast. For T1 weighting, a short effective echo time is chosen and the pulse sequence is triggered every heart beat to keep the repetition time short. For T2 weighting, a long effective echo time is used and the pulse sequence is triggered only every two or three heart beats to achieve a long repetition time. Frequency selective fat suppression may also be applied to suppress the signal from fat if required. The time delay (TI) after the Black blood preparation scheme is automatically calculated by the MR system software to provide the best suppression of signal from blood. This depends on the TR of the pulse sequence which is determined by the patient's heart rate and the number of heart beats between each trigger pulse.

A common problem with this pulse sequence is loss of signal from the myocardium due to motion of the re-inverted myocardial tissue out of the image slice between the time of the black-blood preparation scheme and the time of the turbo or fast spin echo data acquisition. This effect can be reduced by increasing the thickness of the slice of tissue that is re-inverted by the second 180° pulse of the black-blood preparation scheme. While the image slice thickness may be typically 6-8 mm, a more appropriate value for the black blood inversion preparation pulse is 20 mm. The exact choice depends on how much displacement of the myocardium there is through the slice and it requires some adjustment depending on the trigger delay, slice orientation and location within the heart.

### Cine Imaging

**Figure 25:**
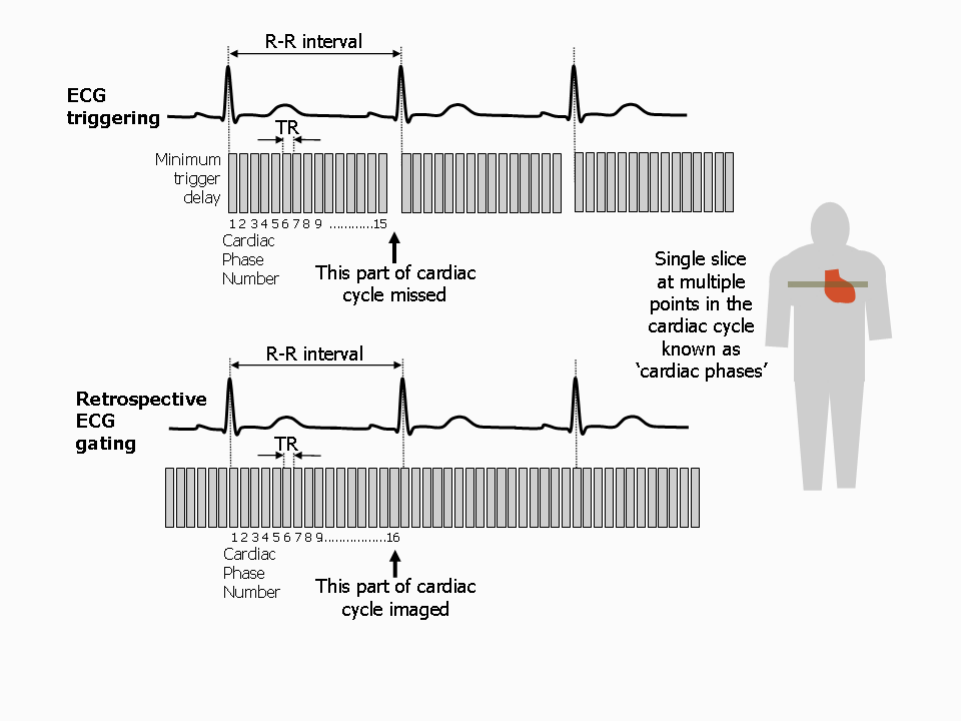
**Cine gradient echo imaging: ECG triggering versus retrospective ECG gating**. Cine imaging is achieved by acquiring data for a single slice location at multiple time points throughout the cardiac cycle. Multiple images are then reconstructed at the corresponding time points, known as cardiac phases. These images are then viewed as a movie to allow the visualisation of cardiac motion and blood flow patterns. ECG triggering (top) initiates the data acquisition of the first cardiac phase immediately after the R-wave and acquires a predetermined number of cardiac phases. The data acquisition must stop before the end of the cardiac cycle to allow detection of the next R-wave. This results in the last part of the cardiac cycle not being imaged. Retrospective ECG gating (bottom) acquires data continuously and records the temporal position of the acquired data relative to the R-wave. The acquisition continues until k-space is filled for a sufficient number of time points and the data is sorted into cardiac phases retrospectively. This approach allows data to be assigned accurately to the end of the cardiac cycle, ensuring the whole of the cardiac cycle is imaged.

Cine imaging requires very short repetition times to be used and therefore can only be achieved using gradient echo-based pulse sequences. It involves the acquisition of data at multiple time points, known as cardiac phases, throughout the cardiac cycle (Figure 25). The trigger delay for the first time point is set to the shortest possible time after the R wave to enable images to be acquired from the beginning of the cardiac cycle. Data acquired within each cardiac phase fills a separate k-space, resulting in the reconstruction of a separate image corresponding to each cardiac phase. The images for all the cardiac phases are then viewed as a movie sequence or cine, allowing functional assessment of the heart, its wall motion and a visual, qualitative assessment of blood flow.

### Triggering versus retrospective gating for cine imaging

For cine imaging, cardiac synchronisation can be performed in either of two ways: ECG triggering or ECG Gating (Figure 25). With ECG triggering the shortest possible trigger delay is used to commence data acquisition immediately after the QRS complex. Data is then acquired for multiple consecutive cardiac phases until nearly at the end of the cardiac cycle. Data acquisition is then stopped until the synchronisation pulse from the next 'R'-wave is received. This method requires the system to estimate an average R-R interval for the patient being imaged (This is either entered by the operator or captured from the ECG trace by the MR system). This is then used to determine the average length of the cardiac cycle over which data can be acquired and therefore how many cardiac phases can be acquired.

A consequence of this approach is that there is a 'blind spot' where no data is acquired at the end of the cardiac cycle while the system waits for the next trigger pulse. This is a disadvantage if imaging of diastolic function or mitral and tricuspid valve function is important. An alternative is to use retrospective ECG gating [23]. Here the pulse sequence runs continuously with a short TR. The synchronisation pulse is used to record when a repetition of the pulse sequence is coincident with the 'R'-wave. The MR signal data from this and subsequent repetitions are then allocated to the corresponding time points in the cardiac cycle at the end of the entire k-space acquisition. With some refinement, retrospective gating can be used successfully when imaging patients with *small *beat-to-beat variations in RR interval. The method acquires data from the whole of each heart beat, so that heart beats of different lengths will have different numbers of data points recorded. At the end of the data acquisition, an average heart beat interval is calculated from the whole acquisition. The time intervals between data points acquired from shorter heart beats are stretched and data from longer heart beats are compressed to fit the average heart beat interval, ensuring that all points in the cardiac cycle are imaged. The use of retrospective gating is essential for applications such as imaging mitral or tricuspid valve function or atrial contraction.

While retrospective gating works well for small beat-to-beat variations in the R-R interval, imaging of patients with large beat to beat variations is problematic. For occasional arrhythmias, there is usually an option for data points acquired from excessively long or short heart beats to be rejected and reacquired. This is known as arrhythmia rejection. In cases where there are many arrhythmias however, rejection of data is not practical and the only options are to revert to a triggered data acquisition if only systolic information is required, or to use a 'real-time' image data acquisition for which ECG synchronisation is not required [24-27]. The latter approach can only be taken at the expense of temporal and spatial resolution.

### Turbo (or Fast) Gradient echo pulse sequences

**Figure 26:**
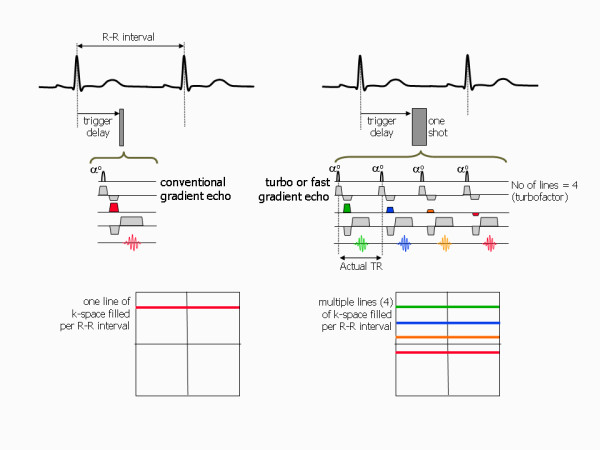
**Accelerated cine imaging with turbo or fast gradient echo**. The conventional gradient echo pulse sequence (left) applies a single low flip angle rf pulse to generate a single gradient echo. One line of k-space is filled each R-R interval for each cardiac phase. The turbo or fast gradient echo pulse sequence (right) rapidly repeats the low flip angle rf pulse to generate multiple gradient echoes. Multiple lines of k-space are filled within each R-R interval by applying a different amplitude of phase encoding gradient to each echo. In this diagram each phase encoding gradient is colour coded corresponding to the line of k-space filled. In this example, four rf pulses are applied to generate four gradient echoes, a parameter setting known as turbofactor= 4 (Philips), no of segments = 4 (Siemens), no of views per segment = 4 (GE). This constitutes one 'shot'. The remaining lines of k-space are filled by acquiring multiple shots over successive heart beats. This provides a fourfold acceleration of the image acquisition time.

Acceleration of cine gradient echo imaging is achieved simply by rapidly repeating the gradient echo pulse sequence a number of times to acquire a number of lines of k-space within each cardiac phase (Figure 26). Each group of k-space lines acquired is known as a shot. This is repeated for each cardiac phase and then for each heart beat, each time acquiring a different group of lines in each successive heart beat until the whole of k-space is filled. This is known as segmented k-space gradient echo imaging, as k-space is segmented into a series of groups of lines [28,29], and is sometimes referred to as multiple shot imaging.

The parameter that defines the number of lines of k-space acquired in each shot is dependent upon the manufacturer as follows:

Philips   turbofactor

Siemens   no of segments*

GE   views per segment

(*Note that on the Siemens interface, a single line of k-space is called a segment). This determines the acceleration factor for a particular pulse sequence. For functional imaging it also determines the length of the acquisition window corresponding to each phase of the cardiac cycle. Increasing the 'turbofactor' decreases the scan time (shortens the length of breath-hold) but increases the acquisition window for each cardiac phase, thus limiting the number of cardiac phases that can be imaged, resulting in a lower cine frame rate or poorer temporal resolution. In order to maximise the number of cardiac phases and minimise the breath-hold period, the ability of an MR system to achieve very short TR values is therefore an advantage.

For breath hold cine gradient echo imaging, this method of accelerated image acquisition can be applied to both spoiled gradient echo and bSSFP pulse sequences commonly used for cardiac imaging. The vendor-specific names for the 'turbo' or 'fast' versions of these sequences are given below:

Fast spoiled gradient echo.

Siemens   TFL   TurboFLASH

Philips   T1-TFE   T1-weighted Turbo Field Echo

GE   FSPGR   Fast SPoiled GRASS

Balanced Steady State Free Precession (bSSFP)

Siemens   Segmented True FISP

Philips BTFE Balanced Turbo Field Echo

GE   FIESTA   Fast Imaging Employing Steady sTate Acquisition

### Functional imaging with turbo or fast cine gradient echo pulse sequences

**Figure 27:**
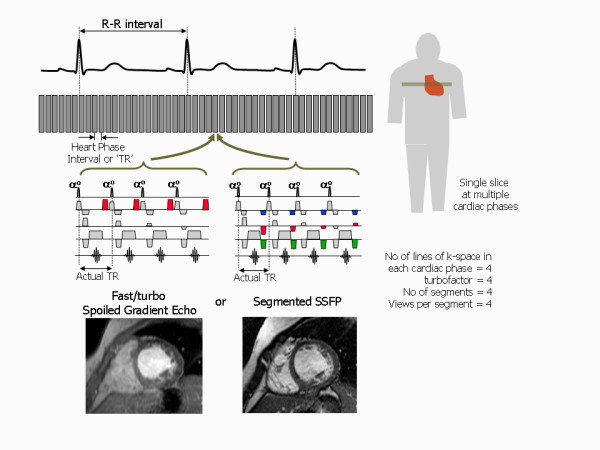
**Bright blood functional cine imaging using retrospectively-gated turbo or fast gradient echo**. Either spoiled gradient echo or balanced SSFP pulse sequences may be used for this application. The number of lines of k-space acquired in each cardiac phase (in this example = 4) determines the acquisition time for this sequence (typically within a single breath-hold period). Increasing the number of lines (the turbofactor, no of segments or no of views per segment) shortens the acquisition time but increases the time between heart phases (the heart phase interval or 'TR'), resulting in poorer temporal resolution. bSSFP pulse sequences can achieve a shorter TR, resulting in shorter breath-hold periods for the same spatial and temporal resolution. The image examples show an end diastolic phase (cardiac phase 1) for spoiled gradient echo (left) and bSSFP (right). Note that the blood signal has more flow dependence for the spoiled gradient echo technique with the LV blood pool (through plane flow) is much brighter than the right ventricle (in-plane flow), whereas for the bSSFP technique they are of equal brightness due to the intrinsic contrast between blood and myocardium based on the T2/T1 ratio. Similarly, the slow-moving blood adjacent to endocardial border is partially saturated in the spoiled gradient echo image, resulting in poorer definition of the endocardial border. This leads to systematic differences in volumetric measurements between the two methods, with the bSSFP techniques yielding larger volumes and smaller ventricular masses.

Imaging of cardiac function, including the assessment of wall motion and volumetric assessment is performed using a 'bright-blood' cine gradient echo technique that acquires images at multiple heart phases which are displayed as a movie. The most common approach to cine imaging is to combine retrospective gating with a turbo or fast gradient echo method (Figure 27). This allows imaging of the entire cardiac cycle within a single breath-hold period. The choice of gradient echo pulse sequence depends on the field strength and the specific application. At 1.5 Tesla the balanced SSFP gradient echo sequence is used for most functional imaging applications and volumetric assessments due to the high intrinsic contrast it achieves between blood and myocardium throughout the cardiac cycle [30-33]. The presence of artefacts initially limited the use of the bSSFP pulse sequence at 3.0T [34], although it is has been used successfully in clinical practice and recent technical advances have indicated that improvements in quality and reliability of this method at 3.0T are possible [35-37]. The spoiled gradient echo pulse sequence is often used for the assessment of valvular disease and flow jets, due to its greater flow sensitivity. It is also often used in preference to the bSSFP technique at 3.0T as it is less prone to artefacts [34,36]. A comparison of clinical applications for spoiled gradient echo and bSSFP techniques is given in Table 2.

**Table 2:** Spoiled gradient echo versus bSSFP cine imaging.

Cine imaging technique	Pulse sequence	Application
Cine gradient echo	Spoiled gradient echo Fast Spoiled GRASS (FSPGR) T1-TFE turboFLASH	Function, qualitative flow assessment, flow jets, regurgitation

Cine SSFP imaging	Balanced SSFP FIESTA Balanced TFE Segmented True FISP	Function, volumetric measurements

A key parameter for cine pulse sequences is the number of lines of k-space acquired within each heart phase (turbofactor, no of segments or number of views per segment). Increasing the value of this parameter shortens the acquisition time, but also increases the time between cardiac phases. This reduces the number of cardiac phases within the cardiac cycle and therefore the cine frame rate or 'temporal resolution' of the image acquisition (the ability to resolve faster motion). 'Real-Time' imaging is achieved by selecting a very high turbofactor [24-27], such that the whole image acquisition is completed in a single cardiac phase in a single heart beat (a single-shot acquisition). Since all the phase encoding steps are acquired in a single heart beat, cardiac synchronisation is not required for real time imaging. The drawback of the high turbofactor is a poor cine frame rate. An acceptable frame rate can only be achieved by reducing the total number of k-space lines acquired. Real time imaging therefore suffers from poor temporal and spatial resolution, although the use of parallel imaging [38-40] can help to preserve spatio-temporal resolution.

## Conclusion

This review has outlined the key physical principles that underlie the most commonly used cardiac MR imaging techniques. The basic principles of MR signal generation and image production have been explained and key imaging parameters have been defined, explaining their influence on image contrast, resolution and acquisition time. It has been shown how fast spin echo and gradient echo imaging techniques can be combined with cardiac synchronisation methods to provide high quality anatomical and functional cine imaging of the heart within a single breath-hold period.

## Competing interests

The author declares that they have no competing interests.

## Authors' contributions

JPR drafted, read, revised and approved the final manuscript.

## Supplementary Material

Additional file 1 **Phase and frequency encoding**. A PowerPoint animation showing steps 2 and 3 in the image formation process. Step 2 applies a magnetic field gradient in the phase encoding direction. This changes the resonant frequency of the spins along this direction, resulting in a phase shift that is related to position in this direction when the gradient is switched off. Step 3 applies a magnetic field gradient in the frequency encoding gradient, resulting in a resonant frequency that is related to position along this direction. The MR signal is measured during step 3 and is the sum of all the frequencies produced. Click here for file
