# Supplementary material for: Cardiovascular magnetic resonance physics for clinicians: part I
Source: J Cardiovasc Magn Reson. 2010 Nov 30;12(1):71. doi: 10.1186/1532-429X-12-71 (PMC3016368; doi:10.1186/1532-429X-12-71)
Supplement: Additional file 1 — Phase and frequency encoding. A PowerPoint animation showing steps 2 and 3 in the image formation process. Step 2 applies a magnetic field gradient in the phase encoding direction. This changes the resonant frequency of the spins along this direction, resulting in a phase shift that is related to position in this direction when the gradient is switched off. Step 3 applies a magnetic field gradient in the frequency encoding gradient, resulting in a resonant frequency that is related to position along this direction. The MR signal is measured during step 3 and is the sum of all the frequencies produced. [file 1532-429X-12-71-S1.PPTX]

## Slide 1
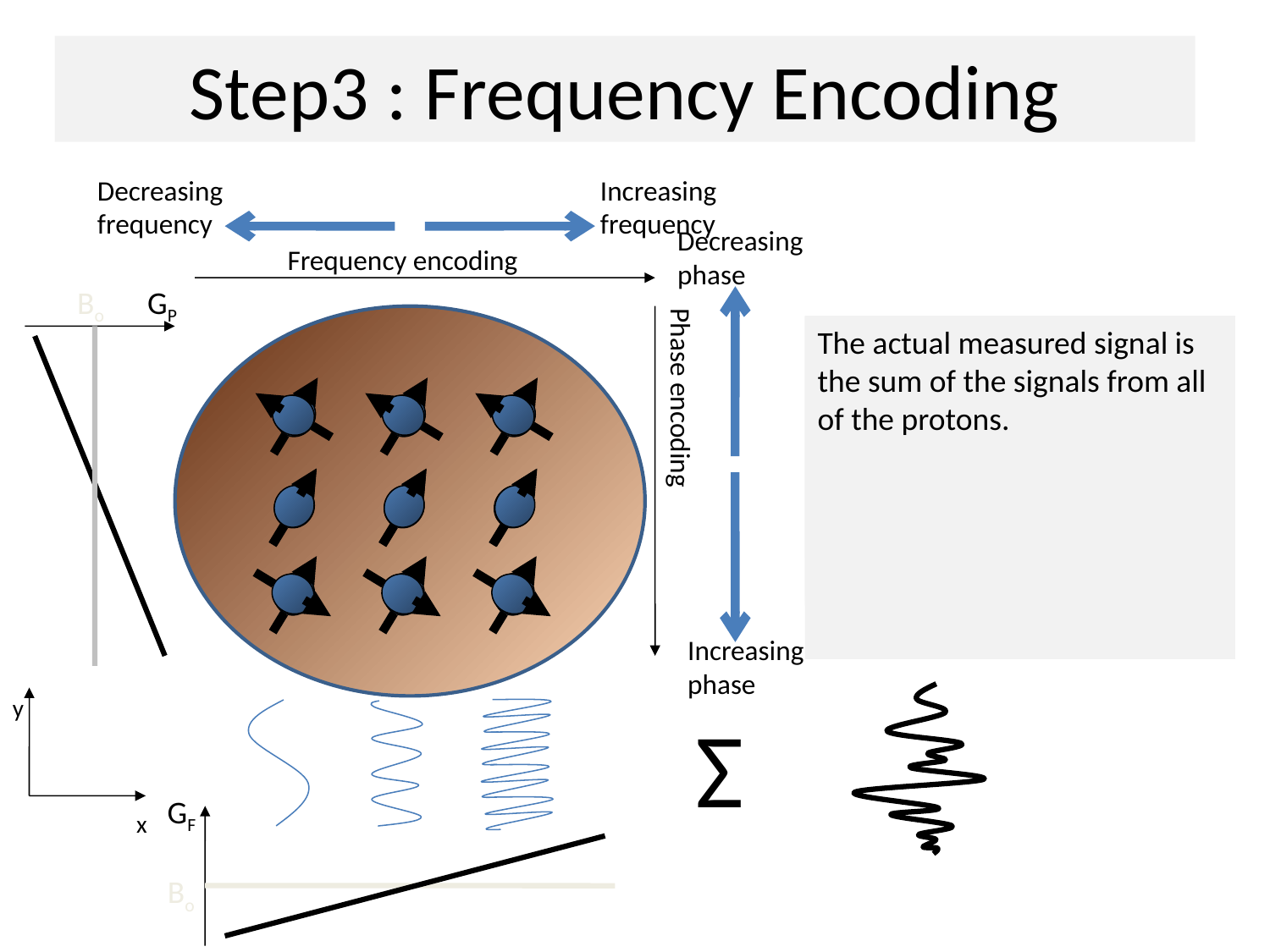

Step2 : Phase Encoding
Step3 : Frequency Encoding
Decreasing frequency
Increasing frequency
Decreasing phase
Increasing phase
Frequency encoding
Bo
GP
Initially all the protons precess in phase with the same frequency, dependent on Bo.
The phase encoding gradient causes the spins to precess at different frequencies for a short time.
When the phase encoding gradient is turned off the protons precess with the same frequency but different phase offsets.
The frequency encoding gradient, is then applied in the x-direction
The frequency encoding gradient causes the protons to precess with different frequencies along the x-direction whilst the signal is read out.
The actual measured signal is the sum of the signals from all of the protons.
Phase encoding
y
x
Σ
GF
Bo
